# Antibodies elicited by the first non-viral prophylactic cancer vaccine show tumor-specificity and immunotherapeutic potential

**DOI:** 10.1038/srep31740

**Published:** 2016-08-22

**Authors:** Jason J. Lohmueller, Shuji Sato, Lana Popova, Isabel M. Chu, Meghan A. Tucker, Roberto Barberena, Gregory M. Innocenti, Mare Cudic, James D. Ham, Wan Cheung Cheung, Roberto D. Polakiewicz, Olivera J. Finn

**Affiliations:** 1 University of Pittsburgh School of Medicine, Department of Immunology, Pittsburgh, PA, USA; 2 Cell Signaling Technology, Inc., Danvers, Massachusetts, USA; 3 Florida Atlantic University, Department of Chemistry and Biochemistry, Boca Raton, Florida, USA; 4 Carnegie Mellon University, Department of Biomedical Engineering, Pittsburgh, PAUSA

## Abstract

MUC1 is a shared tumor antigen expressed on >80% of human cancers. We completed the first prophylactic cancer vaccine clinical trial based on a non-viral antigen, MUC1, in healthy individuals at-risk for colon cancer. This trial provided a unique source of potentially effective and safe immunotherapeutic drugs, fully-human antibodies affinity-matured in a healthy host to a tumor antigen. We purified, cloned, and characterized 13 IgGs specific for several tumor-associated MUC1 epitopes with a wide range of binding affinities. These antibodies bind hypoglycosylated MUC1 on human cancer cell lines and tumor tissues but show no reactivity against fully-glycosylated MUC1 on normal cells and tissues. We found that several antibodies activate complement-mediated cytotoxicity and that T cells carrying chimeric antigen receptors with the antibody variable regions kill MUC1^+^ target cells, express activation markers, and produce interferon gamma. Fully-human and tumor-specific, these antibodies are candidates for further testing and development as immunotherapeutic drugs.

Monoclonal antibodies (mAbs) that specifically bind tumor-associated antigens have been successfully used for cancer immunotherapy^1^. They can target and kill tumor cells directly through complement binding or by interfering with signaling pathways and thus inhibiting tumor growth, or indirectly via mechanisms such as induced phagocytosis or antibody-dependent cell-mediated cytotoxicity (ADCC)^2^,^3^,^4^. They can also be engineered as antibody drug conjugates to specifically deliver radiation or cytotoxic drugs to the tumor bed or to redirect a patient’s T cells to kill tumor cells as parts of bi-specific antibodies or chimeric antigen receptors (CARs)^5^,^6^,^7^.

The MUC1 (Mucin 1) glycoprotein has many characteristics that make it an ideal tumor antigen for cancer immunotherapy approaches. It is overexpressed and abnormally hypo-glycosylated in its extracellular ‘variable number of tandem repeats’ (VNTR) domain on nearly all adenocarcinomas (cancers of the pancreas, lung, breast, colon, prostate and others) as well as on multiple myelomas and some B and T cell lymphomas, accounting for over 80% of human cancers^8^,^9^,^10^,^11^,^12^. Hypo-glycosylation exposes the VNTR peptide core creating tens to hundreds of repeated, cancer-specific epitopes on each MUC1 molecule, which are commonly expressed on various tumor types among most patients^13^. In addition to serving as a tumor marker, abnormally glycosylated MUC1 plays a causative role in tumorigenesis by altering signaling through the EGFR, beta-catenin, NFkB, and p53 pathways^14^,^15^. Importantly, hypoglycosylated MUC1 has also been found to be a target of immunosurveillance in cancer patients. Naturally occurring anti-MUC1 antibodies have been correlated with better disease prognosis and are well-established biomarkers for a variety of cancers^16^,^17^.

We had previously tested MUC1 peptide vaccines composed of the cancer-specific core peptide antigen consisting of 5 copies of the unglycosylated 20mer tandem repeat sequence from the VNTR region, combined with different adjuvants. These vaccines have shown success in eliciting tumor rejection responses in preclinical animal models^18^,^19^. However, they were much less immunogenic when given to late stage cancer patients in Phase I/II trials^20^,^21^,^22^, likely due to the immunosuppressive tumor microenvironment. A clinical trial of one of these vaccines, MUC1 100mer peptide plus Poly-ICLC (Hiltonol), was recently completed in the prophylactic setting in patients at-risk for adenocarcinomas of the colon^23^. It was the first time that a cancer vaccine based on a fully human, tumor-associated abnormal self-antigen was given to healthy individuals. Trial participants were selected on the basis of having a history of colonic polyps that put them at higher risk for colon cancer. Their polyps had not progressed to cancer and thus they were not expected to have developed many immunosuppressive mechanisms found in cancer patients. Nearly half of the vaccinees responded with high titers of antibodies against the MUC1 vaccine peptide and developed strong immune memory. Over the period of observation of more than 5 years, those individuals showed no evidence of adverse effects of the presence of antibodies elicited by the vaccine. This result suggested that these antibodies would also be well-tolerated if transferred to another individual as passive immunotherapy and should have optimal pharmacokinetic properties since they are directly derived from endogenous circulating antibody sequences.

It is well-accepted that fully human antibodies are the safest antibodies, having the lowest chance of inducing unfavorable immune reactions against themselves and having undergone selection and affinity maturation in the full context of human self-antigens^24^. Having the prophylactic vaccine trial as a unique source of human antibodies raised to a tumor-specific antigen and affinity matured in a human host in the absence of cancer, we sought to identify and clone these antibodies and characterize their tumor specificity and range of affinities, in support of their potential as new reagents for cancer immunotherapy. To do so we employed our recently developed proteomics based approach, which combines next-generation sequencing (NGS) and liquid chromatography-tandem mass spectrometry (LC-MS/MS) to identify and isolate circulating antibodies^25^,^26^. Compared to cell-based antibody generation methods or combinatorial display technologies, this platform is highly suitable for isolating monoclonal antibodies from vaccinated donors with high titers of circulating anti-vaccine antibodies, as was demonstrated with a hepatitis B virus vaccine recipient^26^. Availability of matched plasma and B cells from peripheral blood of MUC1 vaccine responders, combined with the well-defined vaccine peptide for affinity purification of antibodies, provided a unique opportunity to isolate and characterize endogenous human anti-MUC1 antibodies elicited by the vaccine.

## Results

### Antibodies elicited by the vaccine react with the MUC1 vaccine peptide

Donors of plasma and B cells for the isolation of antibodies were vaccinated with the MUC1 100mer peptide plus Hiltonol adjuvant at week 0, week 2, week 10, and week 52. Plasma was collected at each time point and screened for vaccine peptide-specific IgG titer by enzyme-linked immunosorbant assay (ELISA). Several recipients responded to the vaccine with high titers of anti-MUC1 IgG^23^. We chose to isolate antibodies from one donor who showed an especially robust memory response to a boost at 1 year following the original vaccination. Both plasma and cryopreserved peripheral blood mononuclear cells (PBMC) from this donor were collected two weeks after the 1-year booster injection. Plasma IgGs exhibited a binding activity to the 100mer MUC1 peptide with a half maximal effective concentration (EC_50_) of approximately 6 μg/ml (Supplementary Fig. 1).

### Isolation and sequencing of vaccine-elicited anti-MUC1 monoclonal antibodies

**Figure 1:**
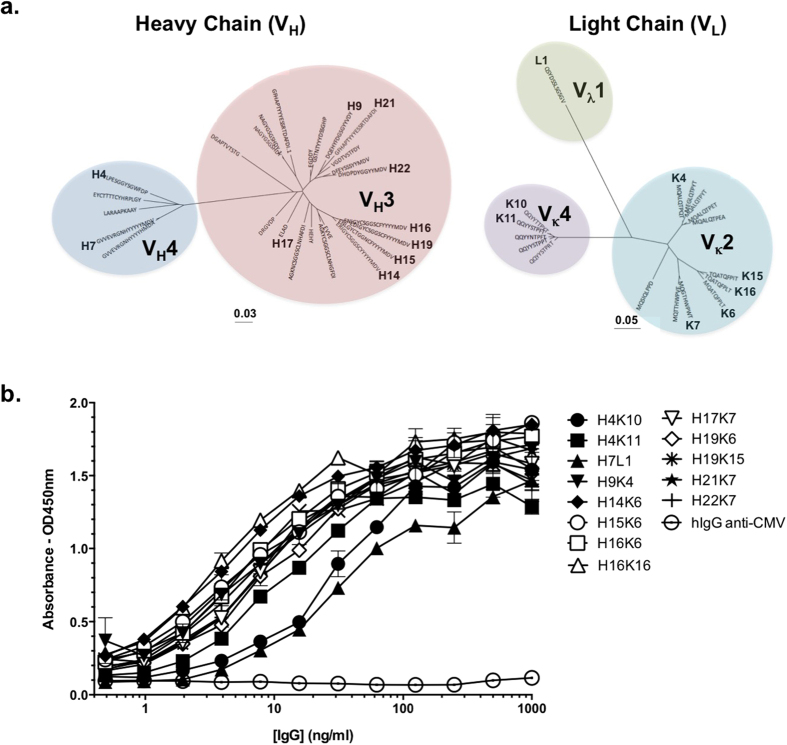
Identification of antibodies specific for the MUC1 100mer vaccine peptide isolated from a vaccinated donor using NGS/LC-MS/MS. (**a**) Phylogenetic dendrogram of variable region sequences. Each unique antibody chain is identified by its CDR3 sequence, and chains that generated active antibodies are indicated with an identifier number next to the CDR sequence. The scale indicates the rate of nucleotide substitution per-site in each tree. The V gene family of each cluster is indicated. (**b**) ELISAs testing antibody binding to immobilized MUC1 100mer vaccine peptide. Assays were performed in triplicate and results are shown as absorbances at 450 nm ± S.D. Human IgG mAb “hIgG anti-CMV”, specific for a glycoprotein from Cytomegalovirus was used as a non-specific antibody binding control^26^.

Vaccine-specific IgGs were affinity-purified from plasma of the vaccine responder using the 100mer MUC1 vaccine peptide conjugated to magnetic beads. The specific activity of the eluted IgG was enriched approximately 1000-fold in binding activity compared to the total IgG from plasma as tested by ELISA (Supplementary Fig. 1). Affinity-purified antibodies were digested with proteases and peptides were then detected by LC-MS/MS. The sequences were mapped to the library of IgG variable region sequences generated by next-generation sequencing (NGS) of cDNA obtained from the donor’s peripheral blood mononuclear cells (PBMC). A total of 416,940 gamma chain, 276,731 kappa chain and 169,788 lambda chain sequences were generated using the 454 deep sequencing platform from cDNA isolated from a total of 13.6 × 10^6^ PBMC. In this manner, variable sequences comprising 26 distinct gamma chain CDR3, 16 distinct kappa chain CDR3 and one lambda chain CDR3 were identified (Fig. 1a). For the gamma chain, the 26 sequences represent 21 distinct clonotypes with predominant usage of VH3 gene, followed by VH4. The light chain sequences were more restricted in their clonotype diversity representing 5 kappa chains and 1 lambda chain clonotypes, including Vκ2, Vκ4 and Vλ1 usage. All permutations of the 26 gamma chains each paired with all 17 light chains were expressed by transient transfection and tested for binding activity on the 100mer peptide by ELISA. We identified 13 distinct monoclonal antibodies that displayed specific binding to the 100mer peptide (Fig. 1b). Based on the gamma chain CDR3 sequences and VDJ usage, these MUC1 vaccine peptide-specific antibodies could be classified into 7 distinct clonotypes (Supplementary Table 1).

### Identification of specific epitopes recognized by vaccine-elicited anti-MUC1 IgGs

To narrow down the specific epitopes on the 100mer MUC1 peptide that individual antibodies recognize, we tested whether antibody binding was prevented by the presence of a sugar on a particular serine or a threonine in the tandem repeat sequence. We performed ELISAs using six 20mer glycopeptides corresponding to the sequence of one repeat, HGVTSAPDTRPAPGSTAPPA, with a GalNAc and/or Galβ1,3GalNAc α-linked to either Thr (positions Thr^4^, Thr^9^, or Thr^16^) or Ser (Ser^5^, or Ser^15^) side chains, as well as an unglycosylated 40mer peptide (HGVTSAPDTRPAPGSTAPPA)_2_, representing two tandem repeats. As controls we used two commercially available MUC1-specific mouse antibodies 3C6 and 4H5, the former being unaffected by MUC1 peptide glycosylation and the latter requiring an unglycosylated threonine in the center of its minimal epitope sequence PDTRP^27^. All antibodies bound the 40mer unglycosylated peptide, but for each antibody, glycosylation of certain serine or threonine residues resulted in the loss of binding (Supplementary Fig. 2). The antibodies could be classified into 3 distinct groups by binding abrogation pattern (Table 1 and Supplementary Fig. 2). Antibodies H14K6, H15K6, H16K6, H16K16, H19K6 and H19K15 share binding specificity with the mouse antibody 4H5, where binding was impeded by glycosylation of the central threonine within the PDTRP sequence. Binding by antibodies H17K7, H21K7 and H22K7 was blocked by glycosylation of the third threonine in the tandem repeat, indicating that these antibodies bind a region that is downstream of the PDTRP epitope. The third class of antibodies comprises H4K10, H4K11, H9K4 and H7L1, all of which only bind the unglycosylated 40mer or the vaccine 100mer peptides but did not bind to any of the 20mer peptides. We postulate that the antibodies in this third group are specific for epitopes formed by the junction of two repeats of the VNTR or to an epitope that only fully forms when two or more repeats are present.

### Anti-MUC1 antibodies exhibit a range of binding affinities

We characterized the binding kinetics of the MUC1 antibodies by surface plasmon resonance (SPR) and found them to have a diverse range of affinities (Table 1). Association and dissociation rates (k_on_ and k_off_) were determined using a Biacore T200 instrument. Two peptides of different lengths were used to determine whether the number of repeats would affect the binding kinetics. Binding kinetics on the 100mer peptide were measured by capturing each antibody in a Fab format to eliminate avidity from the bivalency of a whole IgG, as the 100mer peptide contains multiple copies of a repeated epitope. The 25mer MUC1 peptide was used to measure binding kinetics in the whole IgG format to a single repeat sequence (STAPPAHGVTSAPDTRPAPGSTAPP, representing one repeat plus 5 amino acids from the next repeat)^28^. Fabs derived from all antibodies, except H4K10 and H4K11, each of which showed micromolar affinity to the 25mer, bound the 100mer peptide with affinities in the nanomolar to high picomolar range, placing these antibodies amongst the highest affinity anti-MUC1 antibodies reported^29^,^30^. As the binding kinetics to the 100mer peptide were measured with Fab molecules to minimize avidity effects, a whole IgG binding to cells expressing MUC1 with tens to hundreds of repeated binding sites would be expected to have even greater affinity. When assessing binding kinetics to the 25mer peptide, only antibodies H14K6, H15K6 and H16K6 showed measurable dissociation rates to generate accurate affinities. As expected from the lack of binding to the 20mer peptide from the ELISA results (Supplementary Fig. 2), no binding to the 25mer peptide was detectable for H4K10, H4K11, H7L1 and H9K4 by SPR. For all other antibodies, binding to the 25mer peptide was detectable, however the dissociation rates were too rapid for accurate measurements of affinity.

### Antibodies stain native MUC1 on the surface of cancer cell lines

**Figure 2:**
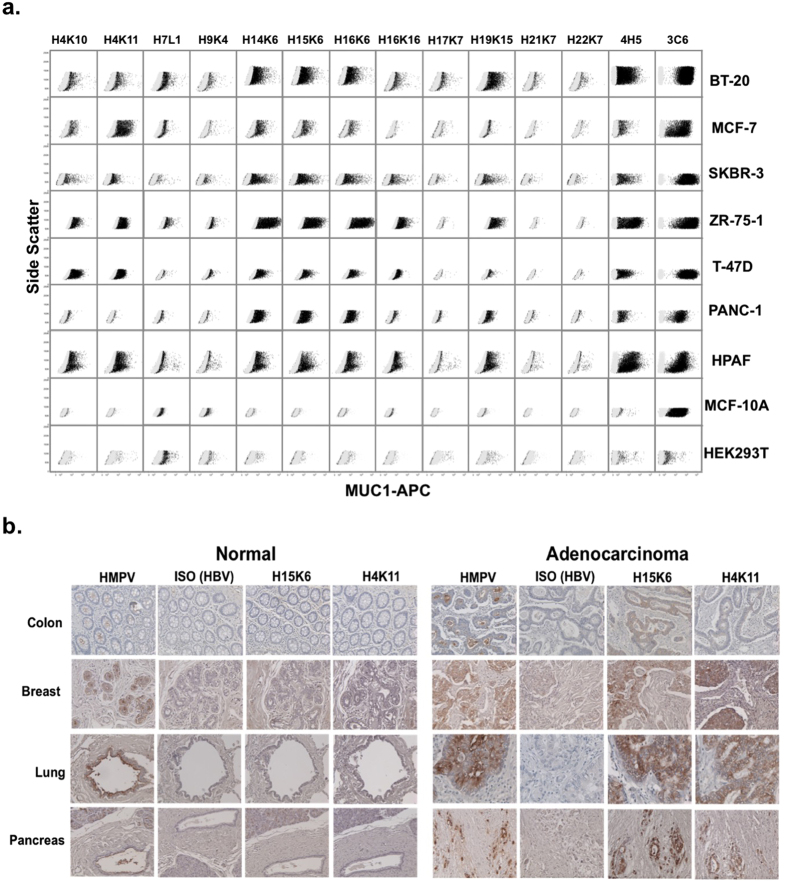
Specific binding of anti-MUC1 antibodies to tumor but not normal cells and tissues. (**a**) Cell lines were stained with the indicated human anti-MUC1 antibodies. Mouse antibodies known to be specific for either the hypo-glycosylated MUC1 (4H5) or all MUC1 (3C6) and a human IgG isotype control antibody were used as positive and negative controls. HEK293T cells do not express MUC1 and are used as a negative control. MCF10A is an immortalized but not transformed cell line that expresses only fully glycosylated (normal) MUC1. Isotype is shown in gray and MUC1 staining in black. (**b**) Paraffin-embedded normal and tumor tissues from human colon, breast, lung and pancreas were stained with mouse antibody HMPV showing total MUC1 expression, with human IgG isotype control antibody as a negative control, and with antibodies H15K6 and H4K11. Images were taken at 20X magnification.

We stained a panel of cancer cell lines with each of the human anti-MUC1 antibodies followed by a dye-conjugated secondary antibody recognizing human IgG Fc. We also stained cells with mouse 3C6 and 4H5 antibodies to show expression of total MUC1 and hypoglycosylated MUC1, respectively. We found that 7 of the 13 antibodies were able to stain the majority of MUC1^+^ cancer cells lines. Importantly, none of these antibodies stained MUC1 on the MCF-10A cell line, an immortalized but not transformed breast epithelial cell line that expresses high levels of fully glycosylated normal MUC1 (Fig. 2a). Antibody binding correlated with the minimal epitope groups determined from the glycopeptide ELISAs (Supplementary Fig. 2), with antibodies predicted to bind to the PDTRP site and junction regions efficiently binding to MUC1 on the surface of cells. We found that two of the MUC1 antibodies (H7L1 and H9K4) showed some staining of HEK293, a cell line that does not express MUC1, indicating non-specific binding, and 3 of the antibodies did not recognize any MUC1 on the cells (H17K7, H21K7 and H22K7), whereas all other antibodies exhibited specificity for hypoglycosylated MUC1 found only on tumor cells.

### Antibodies stain MUC1^+^ cancer but not corresponding normal tissues

To further probe the tumor specificity of the antibodies, we performed immunohistochemical staining of sets of human adenocarcinomas and corresponding normal tissues of the colon, breast, lung and pancreas. The five antibodies that efficiently stained cancer cell lines in Fig. 2a belonged to 2 major heavy chain clonotypes (Supplementary Fig. 2). Thus we chose to stain the tissue sections with antibodies H15K6 and H4K11 representing each of these clonotypes. To identify total MUC1 expression regardless of the glycosylation state, we stained the tissues with HMPV, an anti-MUC1 mouse antibody that binds independently of MUC1 glycosylation (similar in specificity to 3C6 but better for staining paraffin-embedded tissue). We also employed a previously characterized human anti-HBV antibody as an IgG1 isotype control (ISO) for non-specific staining^26^. We observed strong HMPV staining on all adenocarcinoma tissues consistent with overexpression of MUC1, as well as expected weaker staining on the apical surface of the epithelial cells lining the ducts of normal tissues. In contrast, human antibodies H15K6 and H4K11 exhibited strong staining of the tumor tissues, but did not stain the epithelial ducts in normal tissues (Fig. 2b). The only normal tissue staining observed was on the acinar cells of the pancreas. However, the punctate staining pattern was indicative of the antibodies binding to potentially precursor MUC1 molecules in subcellular compartment and not binding on the cell surfaces where the fully processed MUC1 is expected to be (Supplementary Fig. 3).

In addition to these tumor and matched normal sections we also stained a larger series of normal tissues known to express MUC1, including kidney, larynx, uterus, stomach, small intestine, and salivary gland (Supplementary Fig. 4). Each of these tissues contained epithelial ducts that stained strongly with HMPV indicating MUC1 expression, but not with human antibodies H4K11 and H15K6. These antibodies, however, did stain what appeared to be the chief cells of the stomach. Like in pancreatic acinar cells, this staining was also not localized to the cell membrane.

### Effector functions of the MUC1 antibodies

**Figure 3:**
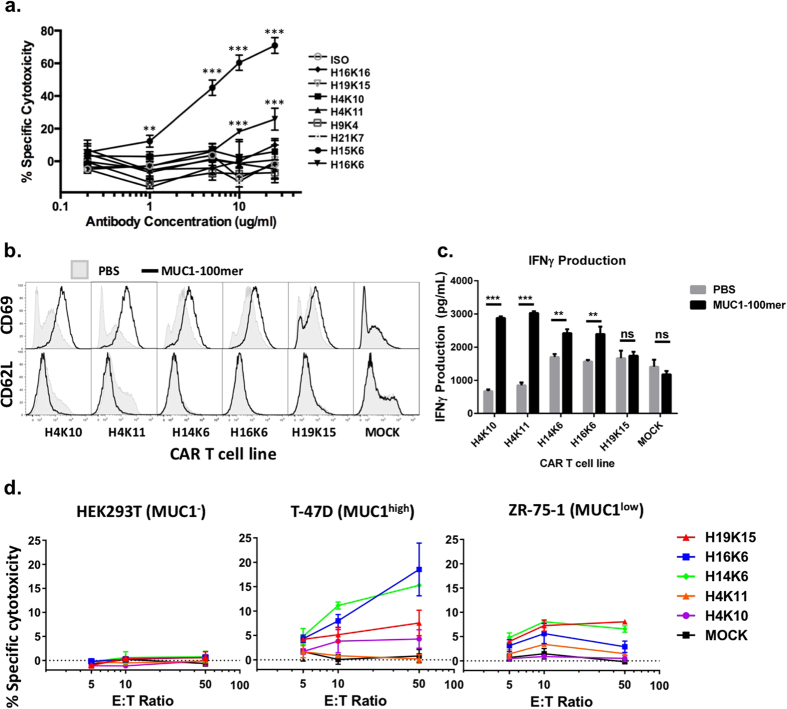
Effector functions of anti-MUC1 antibodies. (**a**) ZR-75-1 breast cancer cells were labeled with calcein-AM dye and co-incubated with baby rabbit complement and various concentrations of each antibody. Assays were performed in triplicate and specific lysis was plotted ± S.D. Pairwise t-tests were performed comparing specific cytotoxicity for anti-MUC1 antibodies and the isotype control antibody (ISO) at each antibody concentration. Statistical significance was determined using the Holm-Sidak method, with α = 0.05. **Indicates p < 0.005 and *** indicates p < 0.001. (**b**) CAR or MOCK transduced T cells with the indicated scFv’s as CAR-targeting domains were incubated for 14hrs in the presence or absence of plate immobilized MUC1 100mer peptide. T cells underwent staining and flow cytometry for T cell activation markers CD69 (up-regulation) and CD62L (down-regulation). (**c**) Supernatants from (**b**) were analyzed for the presence of IFNɣ by ELISA. Assays were performed in triplicate and ELISA data were plotted ± S.D. A pairwise t-test was performed and statistical significance was determined with an α = 0.05. **Indicates p < 0.05 and ***indicates p < 0.001. (**d**) EGFP-expressing target cell lines were co-incubated with CAR T cells at indicated effector:target cell ratios (E:T). Cells were stained with propidium iodide and % cytotoxicity was determined by flow cytometry. Assays were performed in triplicate and data were plotted ± S.D.

We next investigated the ability of the antibodies to perform several known functions that might suggest the types of immunotherapy for which they could be further developed. To test the anti-cancer function of “naked” antibodies we first tested complement-dependent cytotoxicity (CDC). To do so, we co-incubated calcein-AM labeled ZR-75-1 cells with various concentrations of each human anti-MUC1 antibody in the presence of baby rabbit complement. We found that antibodies H15K6 and H16K6 were capable of efficient specific CDC lysis of ZR-75-1 cells as compared to an isotype control antibody and several of the other anti-MUC1 antibodies (Fig. 3a).

Antibody internalization upon binding its cognate surface antigen is a prerequisite for antibody drug conjugate approaches. To assay our anti-MUC1 antibodies for the ability to internalize following binding to cell surface MUC1, we stained ZR-75-1 cells with antibodies H4K11, H14K6, H15K6, H16K6, H19K6 and H19K15 first on ice to observe surface binding, followed by incubation at 37 °C to allow internalization, followed then with fixation and staining with a fluorescently-labeled secondary antibody. We found that each of the antibodies stained the cell surface MUC1 but that very little internalization was observed even after 4 hours of incubation at 37 °C (Supplementary Fig. 5). Investigating further possible internalization of antibody H15K6, we performed confocal imaging and co-stained cells with antibodies against various endocytotic and intracellular markers. We did not observe co-localization with specific endocytic markers such as Caveolin1, Clathrin, Rab5, Rab7 and Rab11, nor with intracellular organelle markers LAMP1 and RCAS1 (Supplementary Fig. 6). Evidence of stable binding to MUC1 on the cell surface but no evidence of internalization, suggests that these antibodies would be better candidates for antibody-radioisotope therapy and bi-specific antibody therapy rather than an antibody-drug conjugate approach.

Next, we tested whether the variable regions of the anti-MUC1 antibodies could be used as components of chimeric antigen receptors (CARs) to retarget effector T cell functions to be MUC1-specific. We constructed lentiviral expression vectors encoding CARs using the variable regions of antibodies H4K10, H4K11, H14K6, H16K6, and H19K15 as antigen binding regions. The constructed CARs were of the 3^rd^ generation format comprising the CD28 and OX40 co-signaling domains in addition to the CD3zeta TCR signaling domain as depicted in Supplementary Fig. 7. We transduced primary T cells with these constructs and confirmed CAR expression by staining of the extracellular IgG4 linker domain (Supplementary Fig. 8). Additionally, we found that 4 out of 5 CARs could be stained using the MUC1-FITC peptide by flow cytometry further indicating that the scFvs of these CARs are functional (Supplementary Fig. 8). We then incubated the CAR^+^ and CAR^−^ primary T cells on plates coated with MUC1 100-mer peptide or PBS and observed MUC1-specific regulation of T cell activation markers (CD69 and CD62L) for all CARs by flow cytometry and production of IFNγ of 4/5 CARs by ELISA (Fig. 3b,c). Finally, we assayed the CAR T cells for their ability to specifically lyse MUC1^+^ cancer cells by co-incubating CAR^+^ primary cells at various concentrations with ZR-75-1(MUC1^low^), T-47D (MUC1^high^) or HEK293T (MUC1^−^) target cells. We observed significant target cell lysis for 3/5 of the CARs - H14K6, H16K6 and H19K15 compared to untransduced ‘MOCK’ cells. The observed cytotoxicity levels were between 5–19% for T-47D (MUC1^high^) and 3–9% for ZR-75-1(MUC1^low^) cells. These values correspond to 9–33% and 8–30% of the hypoglycosylated MUC1^+^ population respectively, as estimated by H15K6 antibody staining (Supplementary Fig. 9). Furthermore, no significant lysis of HEK293T (MUC1^−^) cells was observed (Fig. 3d).

## Discussion

The panel of new human anti-MUC1 antibodies isolated from a MUC1 peptide-vaccinated healthy individual characterized in this study showed exquisite tumor specificity *in vitro* as well as *in vivo* safety in the donor from whom they were derived. These antibodies have undergone selection and affinity maturation in the human host and would be expected to be especially safe without off-target reactivity to other human antigens or MUC1 present on normal tissues. None of the patients who responded to the vaccine (including the donor from whom the antibodies were isolated) had any detectable adverse effects of the antibodies over the 5-year study period^23^. This result is not surprising considering that our analyses of antibody reactivity on cancer cells and human tissue samples showed selective binding to MUC1 on tumor cells only. Being already fully human, these antibodies will not require additional engineering to “humanize” them, a process that could potentially induce changes compromising their tumor specificity and safety^31^. There have been limited attempts to use anti-MUC1 antibodies in therapy as very few mouse antibodies have been identified with tumor specificity. Due to safety concerns, only patients with very advanced disease have been treated and the treatment was largely shown to be of little effect^32^,^33^,^34^,^35^,^36^. One of the key reasons identified for low efficacy has been the elicitation of the human anti-mouse antibody (HAMA) response, further highlighting the importance of having fully-human antibodies^37^,^38^. The potential negative consequences of HAMA for CAR therapy have also been documented^39^,^40^,^41^. Thus our antibodies may be optimal candidates for further development into immunotherapeutic antibodies and CARs on T cells. Our first attempts on the construction and characterization of MUC1 CAR T cells using variable regions of five of the antibodies, showed that they could be activated by MUC1 peptide to produce IFNγ, and lyse tumor cells.

We were especially gratified to find that the highest affinity antibodies were directed against the PDTRP epitope sequence. Most mouse anti-MUC1 antibodies that preferentially recognize MUC1 on tumor cells react with this epitope^27^. We previously determined that in sera of cancer patients with MUC1^+^ tumors, there are anti-MUC1 antibodies specific for this sequence^42^. Furthermore, we previously published isolation and characterization of a MUC1-specific T cell receptor (TCR) from a MUC1-specific human T cell line that also reacts with the PDTRP sequence^43^,^44^. In order to understand the immune-dominance of this sequence, we previously analyzed the structure of the MUC1 VNTR and determined that in the absence of glycosylation, this sequence forms a knob-like structure in each repeat to which antibodies can bind with high avidity^45^. The unglycosylated 100mer vaccine peptide has five PDTRP sequences and the potential to form five immunodominant knobs. From the repertoire of MUC1 vaccine-specific antibodies that we isolated, it is clear that this epitope is highly immunogenic in humans as well as being highly tumor-specific in its expression.

Having a panel of antibodies with diverse binding kinetics and epitopes is highly advantageous for different therapeutic modalities. For antibody-mediated therapies, such as CDC, bi-specific antibodies or other forms of antibody-induced cancer cell death, high affinity mAbs might perform better. Indeed, the two antibodies that were capable of performing CDC in our assays are amongst the highest affinity antibodies we isolated. However, having antibodies that bind at lower affinities could be also useful for applications such as CAR T cell therapies for which it may be beneficial for receptors to mimic the micromolar to high nanomolar affinities of native T cell receptors (TCRs)^46^. Thus, we postulated that the lower affinity antibodies H4K11 and H4K10, and H19K15 may be even better candidates than the higher affinity antibodies (H14K6, H15K6 and H16K6) for CAR development. While we found that T cells carrying CARs corresponding to antibodies H4K10 and H4K11 are most strongly induced to produce IFNγ, we found that only the H19K15 and other CARs with higher affinity domains (H14K6 and H16K6) were able to lyse target cells in our assays. We are continuing to explore whether preferential cytokine production versus cytolytic activity is a feature of a specific CAR affinity or due to a selective expansion of certain T cell populations in culture.

We were surprised to see low or no internalization of our antibodies after binding MUC1 on the tumor cell surface. MUC1 is a molecule that spontaneously internalizes and it appears that antibody binding interferes with this process^47^. This might not be the case with all antibodies as there are reports of MUC1 internalization post-antibody binding^48^. While the antibodies we describe here do not internalize, they could still be used to deliver a therapeutic load to tumor cells, owing to their exquisite specificity for tumor MUC1.

In addition to highlighting the potential of these antibodies as future immunotherapeutics, detailed characterization of the humoral response to the MUC1 vaccine in a healthy individual aids in the overall analysis of a MUC1 prophylactic cancer vaccine potential. Clearly the vaccine was capable of inducing a high affinity polyclonal memory IgG response that is highly tumor-specific and safe. This is exactly what is required of a prophylactic vaccine that would be given to individuals with an increased risk for cancer. The clinical trial that led to the identification of these antibodies was the first in the world prophylactic cancer vaccine trial targeting a non-viral antigen, and this paper is the first to examine and mine the post-vaccination repertoire of a healthy individual vaccinated with a tumor antigen that is an abnormally expressed self-antigen. This work also shows that the proteomic approach we employed was capable of closely recapitulating the circulating immune repertoire raised by the cancer vaccine^25^,^26^. As hypoglycosylated MUC1 is such a prevalent tumor-specific antigen found on a wide variety of human tumors, further development of these and additional antibodies that are being generated using our approach into cancer immunotherapeutic reagents has the potential to aid in the treatment of a very large number of cancer patients.

## Methods

### Anti-MUC1 monoclonal antibody isolation and identification

Serum and B cells from vaccinated human donors were collected as part of clinical trial registered at clinicaltrials.gov (NCT-007773097) with IRB approval of all experimental protocols (IRB# IRB0411047). The vaccine consisted of the 100aa-long peptide composed of 5 20aa-long tandem repeats from the MUC1 VNTR region. For monoclonal antibody identification, total serum IgG was first affinity purified on protein G sepharose beads from 5ml of plasma of a single donor from post 1 year boost blood draw^23^. MUC1 specific IgG was further affinity purified using the MUC1 100mer vaccine peptide conjugated to magnetic beads. The affinity-purified IgG was digested with proteases then analyzed by LC-MS/MS using the LTQ Orbitrap Velos-Elite (Thermo-Fisher Scientific). 454 FLX platform with Titanium chemistry (Roche) was used for the generation of the reference sequence database from 13 million total PBMC obtained from the post-boost time point from the same donor and the variable sequences of gamma, kappa, and lambda chains were mapped to the variable region NGS database.

### Monoclonal anti-MUC1 antibody production

Identified gamma, kappa, and lambda chain variable regions were cloned into mammalian expression plasmids that contained human immunoglobulin IgG1 constant regions and expressed in HEK293 cells as previously described^25^,^26^. MUC1-specific binding was assayed as described below.

### MUC1 peptide ELISA

High binding 96-well ELISA plates (Costar) were coated with various MUC1 peptides by incubating 50 μl/well of peptide at 2 μg/ml in carbonate buffer at 37 °C for 2 hours. Free binding sites on the plate were blocked by addition of 300 μl/well of 5% fat free powdered milk diluted in 1X PBS with 0.1% tween 20 (MPBS-Tween) and incubation at 37 °C for 1 hour. Primary antibody or plasma was diluted in MPBS-Tween, 50 μl/well was added to the plates, which were then incubated at 37 °C for 2 hours. The wells were rinsed with PBS-Tween, then HRP-conjugated goat anti-human IgG antibody (SouthernBiotech) was added followed by addition of tetramethylbenzidine substrate and stop solution (Cell Signaling Technology). Signal level was measured by absorbance at 450 nm.

### MUC1 glycopeptide synthesis

MUC1 glycopeptide analogs were synthesized according to methods described in ref.^49^. The resulting glycopeptides were cleaved from the resin using thioanisole-water-trifluoroacetic acid (5:5:90) for 2 hours. Deacetylation of the sugar hydroxyl groups was accomplished by a treatment with 0.01 M NaOH for 15 minutes. All synthesized MUC1 glycopeptides were purified by reversed-phase high-performance liquid chromatography (RP-HPLC) on a 1260 Infinity Agilent Technologies liquid chromatography system with a Grace Vydac monomeric C_18_ column (250 × 22 mm, 10 μm, 120 Å) at a flow rate of 10.0 mL/min. Eluents were 0.1% TFA in water (A) and 0.1% TFA in acetonitrile (B). The elution gradient was 2% B for the first 5 minutes followed by 0–50% B in 80 min. Detection was at λ = 214 nm. Fractions were analyzed by matrix-assisted laser desorption/ionization time-of-flight mass spectrometry (MALDI-TOF MS) and by analytical RP-HPLC. Analytical RP-HPLC was performed on a 1260 Infinity Agilent Technologies liquid chromatograph equipped with a Grace Vydac monomeric C_18_ monomeric column (250 × 4.6 mm, 5 μm, 120 Å). Eluents were 0.1% TFA in water (A) and 0.1% TFA in acetonitrile (B). The elution gradient was 2–30% B in 20 minutes with a flow rate of 1.0 mL/min. Detection was at λ = 214 nm. MALDI-TOF MS was performed on Voyager MALDI-TOF-DE^TM^ STR mass spectrometer (Applied Biosystems, Foster City, CA) using α-cyano-4-hydroxycinnamic acid matrix. The peptides were dialyzed against water to remove all salt content by using Spectra/Por Float-A-Lyzer with cellulose ester membrane MWCO: 0.1–0.5 kDa.

### MUC1 glycopeptide ELISAs

ELISAs were carried out using a series of 20mer peptides corresponding to a single MUC1 VNTR repeat that has been glycosylated at one or more serine and threonine residues. Each plate was coated with 5 μg of glycopeptides or peptide controls, washed in 1X PBS with 0.1% tween 20 (MPBS-Tween), blocked with 2% BSA, and then incubated with 5 μg/ml of each antibody. The plates were again washed and incubated with an anti-human IgG-HRP conjugated secondary antibody. After another wash step the tetramethylbenzidine substrate reagent was added and after 15 minutes the reaction was stopped using 1 M H_2_SO_4_ stop solution.

### Cell culture and flow cytometry

Human tumor cell lines PANC-1 (pancreatic duct epithelial carcinoma), ZR-75-1 (mammary gland ductal carcinoma), BT-20 (mammary gland carcinoma) MCF-7 (mammary gland adenocarcinoma), MCF-10A (immortalized non-tumorigenic breast epithelium), T-47D (mammary gland ductal carcinoma), HPAF (pancreatic adenocarcinoma), and SKBR-3 (mammary gland adenocarcinoma) were obtained from American Type Culture Collection (ATCC) and cultured at 37 °C in RPMI medium supplemented with 1X MEM amino acids solution, 10 mM Sodium Pyruvate, 10% fetal bovine serum (FBS) and Penicillin-Streptomycin (Life Technologies). HEK293T (human embryonic kidney) cells (ATCC) were cultured at 37 °C in DMEM supplemented with 10% FBS, and Penicillin-Streptomycin.

For flow cytometry, cells were stained using 25 μg/ml of each human anti-MUC1 antibody diluted in flow cytometry buffer (PBS +1% FBS), for 30 minutes at 4 °C followed by two washes with flow cytometry buffer. Cells were then stained with an APC-conjugated F(ab’)_2_ fragment specific to human IgG (Jackson Immunoresearch) and washed twice using flow cytometry buffer. For mouse antibodies 4H5 and 3C6, cells were stained with 1ug/ml of primary unconjugated antibody followed by APC-Cy7 conjugated anti-mouse IgG antibody (eBioscience). Live cells were gated based on forward and side scatter and APC (human) or APC-7 (mouse) fluorescences were measured and plotted against side scatter. 50,000 total events were recorded per sample.

### Determination of antibody binding kinetics

Binding kinetics of human anti-MUC1 antibodies to MUC1 25mer and 100mer peptides were determined using the Biacore T200 platform (GE Healthcare). To assay binding to the 25mer peptide STAPPAHGVTSAPDTRPAPGSTAPP, whole anti-MUC1 IgG was immobilized as a ligand to a CM5 chip coated with anti-human IgG capture antibody, and serially diluted 25mer peptide was used as the analyte. To assay binding to the 100mer peptide, each antibody was first processed into Fab format by digestion with IdeS protease (Genovis) to eliminate crosslinking on tandemly repeated epitopes, followed by partial reduction with β-mercaptoethanol, as described in the manufacturer’s protocol. Each Fab was immobilized at low density (RU < 50) as ligand on a sensor chip CM5 (GE Healthcare) coated with anti-human Fab capture kit, and serially diluted 100mer peptide was used as the analyte. The kinetic analysis was done with the Biacore kinetics analysis software where the fitted curves for binding of the ligand to analyte were modeled as a 1:1 interaction.

### Cell immunofluorescence staining and internalization assays

Binding of anti-MUC1 antibodies to MUC1-expressing cell line ZR-75-1 grown in RPMI medium with 10% fetal bovine serum (FBS) was visualized by immunofluorescence by incubating cells with 25 μg/ml of antibody, diluted in PBS, for 1 hour on ice, after which the cells were rinsed with PBS and the incubation temperature was shifted to 37 °C. 4 hours later, cells were fixed with 4% formaldehyde in PBS, followed by 3 washes with PBS. Anti-MUC1 antibodies were detected with Alexa 488-conjugated goat anti-human secondary antibody (Life Technologies) and visualized using the Cellomics ArrayScan VTI (Cellomics). Binding of anti-MUC1 antibody H15K6 to ZR-75-1 to visualize potential internalization by confocal immunofluorescence microscopy was accomplished as follows: trypsinized cells were seeded on 8-chamber polystyrene vessel glass slides (BD Falcon) at approximately 30% confluence and were cultured for 48 hours prior to incubation with anti-MUC1 antibody. At the time of antibody addition, slides with cells were pre-chilled on ice for 15 minutes then incubated with 25 μg/ml of antibody diluted in ice-cold RPMI with 10% FBS for 1 hour on ice, after which the cells were rinsed with PBS, then incubated at 37 °C in RPMI with 10% FBS for 15 minutes, 30 minutes, 1 hour, 2 hours and 4 hours. At the end of each 37 °C incubation time, cells were fixed and permeabilized with 4% formaldehyde in PBS, followed by a wash with PBS. Rabbit monoclonal antibodies specific to Caveolin1, Clathrin, Rab5, Rab7, Rab11, LAMP1 and RCAS1 (Cell Signaling Technology) were used to detect organelles following the manufacturer’s recommended protocol to detect intracellular organelle localization after fixing and permeabilization of cells. Anti-MUC1 antibodies were detected with Alexa488-conjugated goat anti-human secondary antibody (Life Technologies) and organelle-specific antibodies were detected with Alexa555-conjugated anti-rabbit IgG secondary antibody (Cell Signaling Technology) and visualized by a Nikon C1 confocal microscope (Nikon).

### Immunohistochemical staining of human tissues

All experiments were performed on de-identified human paraffin-embedded tissues which fulfill basic exempt criteria 45 CFR 46.101(b)(4) in accordance with the University of Pittsburgh IRB guidelines. Tissue types and catalogue numbers are listed in Supplementary Table 2. Tissue sections were deparaffinized with a series of three xylene and ethanol washes. Antigen retrieval was performed by microwaving in 0.6 M citrate buffer. All tissues except colon were stained with HMPV antibody (SantaCruz) at a dilution of 1:300 or with a 1:300 dilution of a 1 mg/ml stock of antibodies H4K11, H15K6, or the human IgG isotype control antibody specific for an HBV protein^26^. Colon tissues were stained with a 1:50 antibody dilutions. Tissues stained with human antibodies and mouse antibodies were then stained with HRP conjugated anti-human IgG (SantaCruz) or anti-mouse IgG (SantaCruz) secondary antibodies, respectively. Stained slides were imaged using an Olympus BX40 microscope and Leica DFC 420 camera at 20X magnification.

### Complement dependent cytotoxicity (CDC) assay

ZR-75-1 tumor cells were labeled using 10 μM calcein AM (Life Technologies) at a 2 million cells/ml in complete RPMI media for 30 minutes at 37 °C and washed twice with PBS. Cells were then resuspended in fresh medium and plated at 10,000 cells per well in a 96 well plate. Next, 15 μL of ice-cold baby rabbit complement (Cedarlane Labs) was added to each well followed by the addition of the antibodies at various concentrations in triplicate. Cells were then incubated with complement and antibodies for 6 hours. Triton X was added to ‘maximum lysis’ positive control wells 30 minutes prior to the assay. Finally, 50 μL of cell media for each well was transferred to a new plate and fluorescence was read at 488 nm. Specific cytotoxicity was calculated by the equation: (experimental antibody fluorescence − complement only fluorescence)/(max fluorescence − complement only fluorescence).

### DNA cloning and lentivirus production

The CAR coding regions listed in Supplementary Table 3. were cloned into the pSICO transfer vector backbone (Addgene) containing the EF1-alpha promoter, pSICO-EF1 (see Supplementary Table 3), using Gibson Assembly cloning. To generate virus, HEK293T cells were transfected with pVSV-G (VSV glycoprotein expression plasmid), pLP2 (Rev expression plasmid), pLP1 (Gag/Pol expression plasmid), and a pSICO-EF1-CAR transfer plasmid using calcium phosphate transfection. At 16 hours post-transfection cells were washed with PBS and fed with complete DMEM media containing 6 mM sodium butyrate (Sigma Aldrich). After 24 hours the viral supernatants were collected and cells were given fresh media with sodium butyrate. Supernatants were again collected after another 24 hours. Collected supernatants were then combined and filtered through a 45 μm vacuum filter. Viral particles were concentrated by ultracentrifugation for 1.5 hours at 24,500 rpm, and viral pellets were re-suspended in 0.05 ml of supplemented RPMI medium and frozen at −80 °C.

### Culture, transduction, and expansion of CAR^+^ human T cells

All experiments were performed on PBMC isolated from de-identified human Buffy Coat samples purchased from the Pittsburgh Central Blood Bank fulfilling the basic exempt criteria 45 CFR 46.101(b)(4) in accordance with the University of Pittsburgh IRB guidelines. Human T cells were cultured in supplemented RPMI media as described for cell lines above however, 10% Human AB serum (Gemini Bio Products) was used instead of FBS, and the media was further supplemented with 100 U/ml human IL-2 IS (Milltenyi Biotec). PBMC were isolated from Buffy Coat from healthy volunteer donors using Ficoll centrifugation and total human T cells were isolated using the Pan T cell isolation kit (Milltenyi). T cells were then stimulated and expanded using the Human T cell Activation/Expansion kit (Milltenyi). For transduction, two days after the initial addition of stimulation beads, lentivirus was added to cells at an MOI of 10–50 in the presence of 6 ug/ml of DEAE-dextran (Sigma Aldrich). After 24 hours cells were washed and resuspended in fresh T cell media containing 100 U/ml IL-2. After an additional 12 days of stimulation and expansion, activation beads were removed from the cells, and the CAR^+^ cells were flow-sorted by TagBFP expression. To obtain sufficient numbers of cells for experiments, sorted CAR^+^ cells then underwent an additional cycle of stimulation and bead removal at day 14 post stimulation.

### MUC1 CAR T cell activation and IFNγ assays

High protein binding 96 well flat bottom plates (Corning) were coated with 10 ug/ml of MUC1-100mer peptide in PBS or PBS alone for 2 hrs at 37 °C and washed 2 times with PBS. 100,000 CAR^+^ or mock-transduced primary T cells were incubated on the plate for 14 hrs. After incubation cells were stained with antibodies against T cell activation markers CD69-PE (BD Biosciences) and CD62L (BD Biosciences) and were evaluated for marker expression by flow cytometry. Supernatants from these cell cultures were also collected and analyzed for the presence of IFNɣ by ELISA (BioLegend). Assays were performed in triplicate and average IFN*γ* production was plotted along with standard deviation.

### CAR^+^ T cell lysis assay

EGFP-expressing target cells were re-suspended in human T cell media containing 100 U/ml IL-2 and plated at 10,000 cells per well in 50 ul in a 96 well V-bottom plate. Next, 50 uL of CAR^+^ primary human T cells were added at E:T ratios of 5:1, 10:1, or 50:1. Plates underwent a quick spin to collect cells at the bottom of the wells and then incubated at 37 °C for 4 hours. To identify lysed cells, samples were analyzed by flow cytometry. One minute prior to running each sample, 1 ul of 50 ug/ml propidium iodide (BD Biosciences) was added per 100 ul of sample. Target cells were identified by EGFP expression and lysed target cells were identified by propidium iodide staining. Specific cytotoxicity was calculated by the equation: 100 * (% experimental lysis–% target-only lysis)/(100–% target-only lysis).

## Additional Information

**How to cite this article**: Lohmueller, J. J. *et al*. Antibodies elicited by the first non-viral prophylactic cancer vaccine show tumor-specificity and immunotherapeutic potential. *Sci. Rep.* **6**, 31740; doi: 10.1038/srep31740 (2016).

## Supplementary Material

Supplementary Information

## Figures and Tables

**Table 1:** CDR3 sequences, binding kinetics and predicted minimal epitope of human anti-MUC1 antibodies.

mAb	CDR3-H	CDR3-L	25mer Affinity–Whole IgG			100mer Affinity–Fab			Predicted Epitope^a^
			k_a_ (M^−1^ s^−1^)	k_d_ (s^−1^)	K_D_ (M)	k_a_ (M^−1^ s^−1^)	k_d_ (s^−1^)	K_D_ (M)	
H4K10	LPESGGYSGWFDP	QQYYNTPFT	No Binding			Steady State*		5.31 × 10^−6^	APPHGVTS
H4K11	LPESGGYSGWFDP	QQYYSTPFT	No Binding			Steady State*		1.57 × 10^−5^	APPHGVTS
H7L1	GVVEVRGNHYYYYYMDV	QSYDSSLSGSGV	No Binding			1.01 × 10^6^	3.33 × 10^−3^	3.31 × 10^−9^	APPHGVTS
H9K4	GSTNTYYYDSSGHP	MQALQTPQT	No Binding			1.02 × 10^4^	2.99 × 10 ^4^	2.92 × 10^−10^	APPHGVTS
H14K6	ERIGYCSGGSCYYYYYMDV	MQATQFPLT	4.53 × 10^4^	1.23 × 10^−3^	2.72 × 10^−8^	2.67 × 10^6^	3.46 × 10^−4^	1.30 × 10^−10^	PD**T**RP
H15K6	ENLGYCTGGNCFYYYYMDV	MQATQFPLT	6.81 × 10^4^	4.89 × 10^−4^	7.19 × 10^−9^	2.04 × 10^6^	5.66 × 10^−4^	2.77 × 10^−10^	PD**T**RP
H16K6	ENIGYCSGGSCFYYYYMDV	MQATQFPLT	6.87 × 10^4^	2.35 × 10^−3^	3.42 × 10^−8^	2.02 × 10^6^	6.41 × 10^−4^	3.17 × 10^−10^	PD**T**RP
H16K16	ENIGYCSGGSCFYYYYMDV	TQATQFPLT	7.47 × 10^4^	k_off_ (Too Fast)	—	1.86 × 10^6^	4.07 × 10^−3^	2.19 × 10^−9^	PD**T**RP
H17K7	ELAD	MQGTHWPWT	8.81 × 10^3^	k_off_ (Too Fast)	—	1.43 × 10^6^	1.54 × 10^−3^	1.08 × 10^−9^	GS**T**APP
H19K6	EDIGYCSGGSCFYYYYMDV	MQATQFPLT	1.05 × 10^6^	k_off_ (Too Fast)	—	1.15 × 10^6^	3.52 × 10^−4^	3.07 × 10^−10^	PD**T**RP
H19K15	EDIGYCSGGSCFYYYYMDV	TQATQFPIT	8.60 × 10^3^	k_off_ (Too Fast)	—	8.17 × 10^5^	4.53 × 10^−4^	5.55 × 10^−10^	PD**T**RP
H21K7	DQEHYFDGSGYYVDY	MQGTHWPWT	1.32 × 10^4^	k_off_ (Too Fast)	—	3.07 × 10^6^	6.62 × 10^−3^	2.16 × 10^−9^	GS**T**APP
H22K7	DHDPDYGGYYMDV	MQGTHWPWT	1.27 × 10^3^	k_off_ (Too Fast)	—	4.89 × 10^5^	2.73 × 10^−3^	5.57 × 10^−9^	GS**T**APP

Biacore assays were performed using whole IgG binding to the MUC1 VNTR 25mer peptide as well as Fab fragments binding to the MUC1 VNTR 100mer. Predicted minimal binding epitopes for the antibodies were characterized by glycopeptide ELISA in Supplementary Fig. 2. CDR3-H, heavy chain CDR3; CDR3-L, light chain CDR3; k_on_, association rate; k_off_, dissociation rate, K_D_, equilibrium dissociation constant.
