# Supplementary material for: Antibodies elicited by the first non-viral prophylactic cancer vaccine show tumor-specificity and immunotherapeutic potential
Source: Sci Rep. 2016 Aug 22;6:31740. doi: 10.1038/srep31740 (PMC4992835; doi:10.1038/srep31740)
Supplement: Supplementary Information [file srep31740-s1.pdf]

Supplementary Information:

Antibodies elicited by the first non-viral prophylactic cancer vaccine show tumor-specificity and immunotherapeutic potential

Jason J. Lohmueller[1,a], Shuji Sato[2,a], Lana Popova[2], Isabel M. Chu[2], Meghan A. Tucker[2], Roberto Barberena[2], Gregory M. Innocenti[2], Mare Cudic[3], James D. Ham[1,4], Wan Cheung Cheung[2,b], Roberto D. Polakiewicz[2,*], Olivera J. Finn[1,*]

Affiliations:
[1]University of Pittsburgh School of Medicine, Department of Immunology, Pittsburgh, PA USA
[2]Cell Signaling Technology, Inc., Danvers, Massachusetts, USA.
[3]Florida Atlantic University, Department of Chemistry and Biochemistry, Boca Raton, Florida, USA.
[4]Carnegie Mellon University, Department of Biomedical Engineering, Pittsburgh, PA.
[a]These authors contributed equally to this work.
[b]Current address: Global Biotherapeutic Technologies, Pfizer, Cambridge, Massachusetts 02139, USA.
[*]To whom correspondence should be addressed.

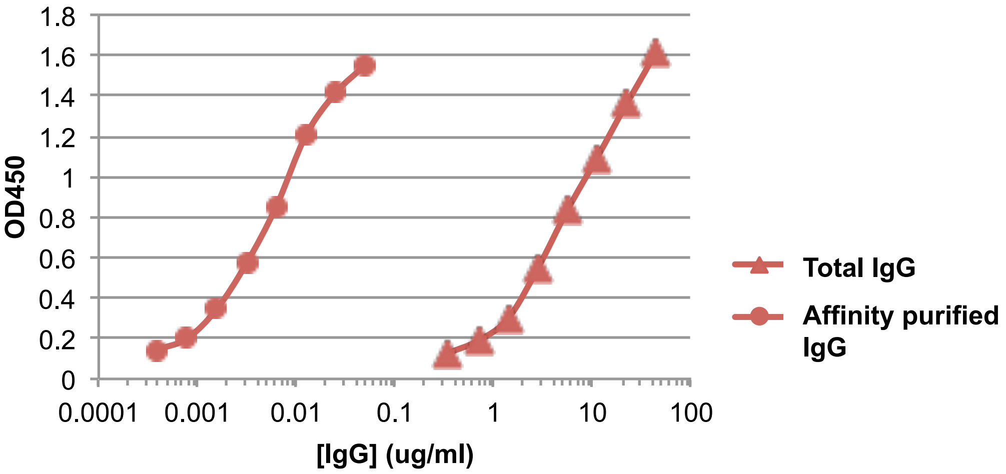

Supplementary Fig. 1. **Specific activity of total plasma IgG and affinity purified IgG from donor binding to 100mer VNTR peptide as determined by ELISA.** Anti-MUC1 vaccine peptide IgG in the plasma of the vaccinee was affinity purified first by protein A purification of total IgG then on 100mer VNTR peptide immobilized to magnetic beads. The specific binding activity of the eluted IgG after protein A purification (total IgG) and after peptide affinity purification (affinity purified IgG) was tested by ELISA.

Supplementary Table 1. Antibody VDJ and VJ usage and the number of somatic hypermutations for the heavy (Hc SHM) and light (Lc SHM) chains of the human anti-MUC1 antibodies.

Mab	VDJ	Hc SHM	VJ	Lc SHM
**H4/K10**	VH4-39;DH1-26;JH5	3	VK4-1;JK3	1
**H4/K11**	VH4-39;DH1-26;JH5	3	VK4-1;JK2	0
**H7/L1**	VH4-39;DH3-10;JH6	5	VL1-40;JL2	1
**H9/K4**	VH3-66;DH3-22;JH3	0	VK2-28;JK2	7
**H14/K6**	VH3-11;DH2-15;JH6	3	VK2-24;JK4	0
**H15/K6**	VH3-11;DH2-8;JH6	3	VK2-24;JK4	0
**H16/K6**	VH3-11;DH2-15;JH6	3	VK2-24;JK4	0
**H16/K16**	VH3-11;DH2-15;JH6	3	VK2-24;JK4	1
**H17/K7**	VH3-74;DH6-6;JH4	1	VK2-30;JK1	3
**H19/K6**	VH3-11;DH2-15;JH6	2	VK2-24;JK4	0
**H19/K15**	VH3-11;DH2-15;JH6	2	VK2-24;JK3	1
**H21/K7**	VH3-66;DH3-22;JH4	1	VK2-30;JK1	3
**H22/K7**	VH3-53;DH3-16;JH6	1	VK2-30;JK1	3

MUC1 VNTR Glycopeptides	Antibodies													
	H4K10	H4K11	H7L1	H9K4	H14K6	H15K6	H16K6	H16K16	H17K7	H19K15	H21K7	H22K7	3C6	4H5
(HGVTSAPDTRPAPGSTAPPA)x2	**3.2**	**3.4**	**3.1**	**3.5**	**3.0**	**2.9**	**2.9**	**3.3**	**2.8**	**3.3**	**3.0**	**3.1**	**2.3**	**2.5**
HGVTSAPDTRPAPGSTAPPA	0.1	0.1	0.1	0.1	**2.1**	**2.0**	**2.1**	**2.2**	**1.9**	**2.4**	**2.1**	**2.1**	**1.5**	**1.9**
HGVTSAPDTRPAPGSTAPPA	0.1	0.1	0.1	0.1	**1.6**	**1.7**	**1.6**	0.1	**2.0**	**1.5**	**2.1**	**2.2**	**2.0**	**1.8**
HGVTSAPDTRPAPGSTAPPA	0.1	0.1	0.1	0.1	**2.1**	**2.4**	**2.1**	**1.3**	**2.1**	**2.7**	**2.1**	**2.2**	**2.0**	**1.8**
HGVTSAPDTRPAPGSTAPPA	0.1	0.1	0.1	0.1	0.1	0.2	0.1	0.1	**1.9**	0.1	**2.1**	**2.3**	**2.4**	0.1
HGVTSAPDTRPAPGSTAPPA	0.1	0.1	0.1	0.1	**2.5**	**2.5**	**2.4**	**2.2**	0.1	**2.5**	0.1	0.1	**1.5**	**1.9**
HGVTSAPDTRPAPGSTAPPA	0.1	0.1	0.1	0.1	0.1	0.1	0.1	0.1	**1.9**	0.1	**2.2**	**2.3**	**2.4**	0.1
HGVTSAPDTRPAPGSTAPPA	0.1	0.1	0.1	0.1	0.1	0.2	0.1	0.1	0.1	0.1	0.1	0.1	**2.4**	0.1
Bovine Serum Albumin (BSA)	0.1	0.1	0.1	0.1	0.1	0.1	0.1	0.1	0.1	0.1	0.1	0.1	0.2	0.1

Supplementary Fig. 2. Binding of antibodies to MUC1 glycopeptides. ELISAs were performed on plates coated with various MUC1 glycopeptides or BSA for each anti-MUC1 antibody. Glycosylated residues on the glycopeptides are highlighted in red. Absorbance values for each peptide/antibody combination are displayed in the heatmap. Two mouse antibodies that bind to either all MUC1 glycoforms (3C6) or only the tumor specific glycoforms (4H5) were used as controls.

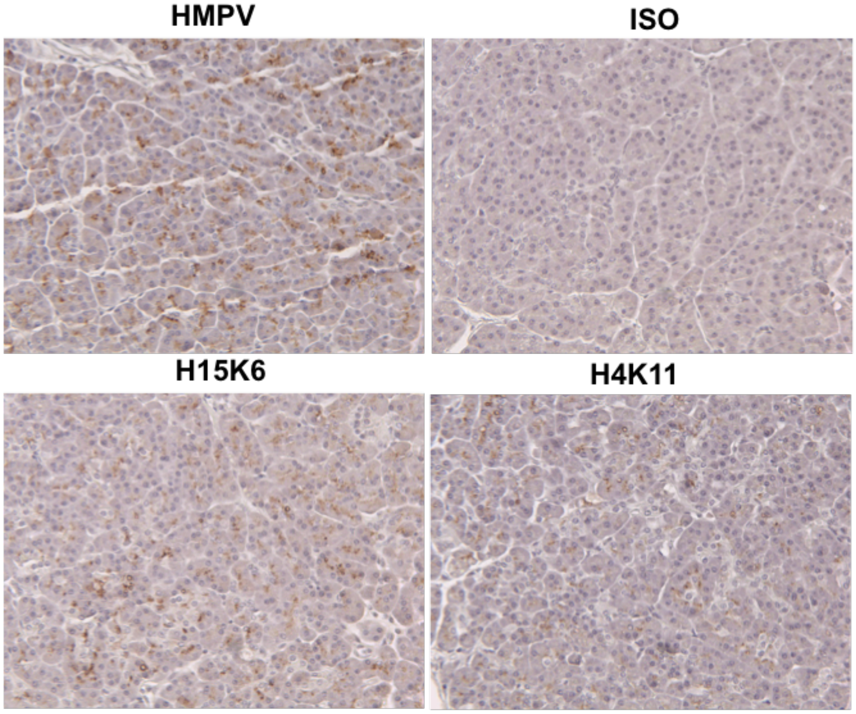

Supplementary Fig. 3. Immunohistochemical staining of pancreatic acinar cells with human anti-MUC1 antibodies. Paraffin-embedded normal pancreas tissue sections were stained with HMPV antibody for total MUC1 expression, human IgG Isotype control antibody as a negative control, and human anti-MUC1 antibodies H15K6, and H4K11. Punctate staining is indicative of subcellular compartment staining. Images taken at 20X magnification.

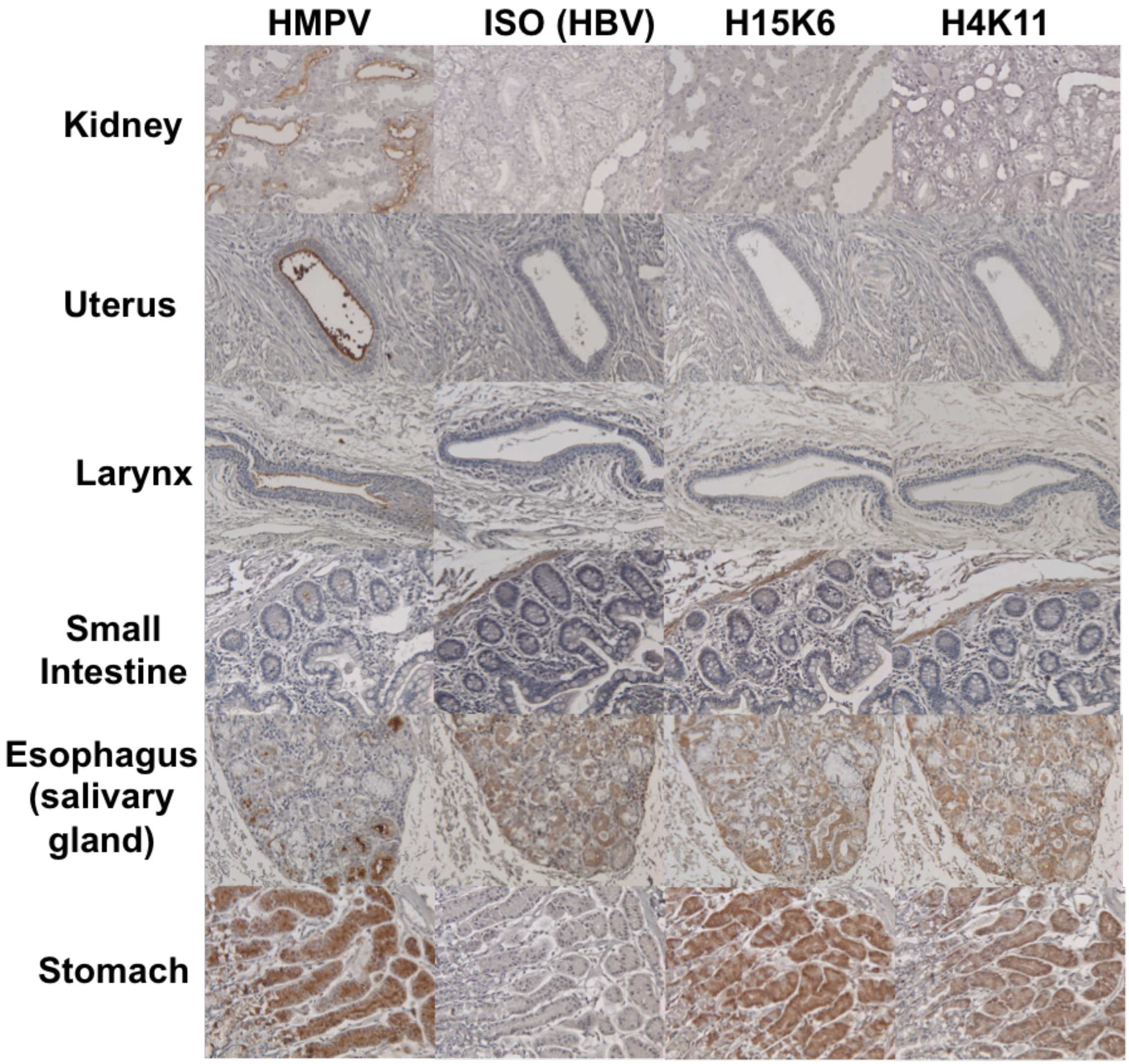

Supplementary Fig. 4. Immunohistochemical staining of paraffin-embedded normal tissue sections with human anti-MUC1 antibodies H15K6 and H4K11. HMPV is a mouse antibody that stains all MUC1 glycoforms (positive control). ISO (HBV) is a human anti-HBV IgG used as an isotype (negative) control. Images taken at 20X magnification.

Supplementary Table 2. Tumor and normal tissue sample information for stained tissue samples.

Catalog ID#	Vendor	Tissue type	Age	Sex
HUCAT171	US BIOMAX	Adenocarcinoma (grade II) of pancreas	54	F
HUCAT171	US BIOMAX	Adenocarcinoma (grade II) of pancreas	54	F
HUCAT226	US BIOMAX	Adenocarcinoma (grade II) of right lung	42	M
HUCAT226	US BIOMAX	Adenocarcinoma (grade II) of right lung	42	M
HUCAT300	US BIOMAX	Human breast invasive ductal carcinoma	47	F
HUCAT300	US BIOMAX	Human breast invasive ductal carcinoma	47	F
HUCAT391	US BIOMAX	Human Kidney Cancer	57	F
HUCAT391	US BIOMAX	Human Kidney Cancer	57	F
HUFPT041	US BIOMAX	Normal Human Esophagus	21	F
HUFPT041	US BIOMAX	Normal Human Esophagus	21	F
HUFPT073	US BIOMAX	Normal Human Kidney Medulla	59	M
HUFPT073	US BIOMAX	Normal Human Kidney Medulla	59	M
HUFPT078	US BIOMAX	Normal Human Body Of Pancreas	63	M
HUFPT078	US BIOMAX	Normal Human Body Of Pancreas	63	M
HUFPT081	US BIOMAX	Normal Human Small Intestine	2	F
HUFPT081	US BIOMAX	Normal Human Small Intestine	2	F
HUFPT096	US BIOMAX	Normal Human Uterus	21	F
HUFPT096	US BIOMAX	Normal Human Uterus	21	F
HUFPT101	US BIOMAX	Normal Human Tongue	21	F
HUFPT101	US BIOMAX	Normal Human Tongue	21	F
HUFPT116	US BIOMAX	Normal Human Larynx	21	F
HUFPT116	US BIOMAX	Normal Human Larynx	21	F
HUFPT130	US BIOMAX	Normal Breast Tissue	35	F
HUFPT130	US BIOMAX	Normal Breast Tissue	35	F
HUFPT178	US BIOMAX	Normal Lung	47	M
HUFPT178	US BIOMAX	Normal Lung	47	M
Colon N.434	CST	Normal Colon	#N/A	#N/A
Colon N.434	CST	Normal Colon	#N/A	#N/A
Colon N.434	CST	Normal Colon	#N/A	#N/A
Colon N.434	CST	Normal Colon	#N/A	#N/A
Colon 433	CST	Colon Adenocarcinoma	#N/A	#N/A
Colon 433	CST	Colon Adenocarcinoma	#N/A	#N/A
Colon 433	CST	Colon Adenocarcinoma	#N/A	#N/A

Colon 433	CST	Colon Adenocarcinoma	#N/A	#N/A
Lung N.947	CST	Normal Lung	#N/A	#N/A
Lung N.947	CST	Normal Lung	#N/A	#N/A
Lung N.947	CST	Normal Lung	#N/A	#N/A
Lung N.947	CST	Normal Lung	#N/A	#N/A
Lung 946	CST	Lung Adenocarcinoma	#N/A	#N/A
Lung 946	CST	Lung Adenocarcinoma	#N/A	#N/A
Lung 946	CST	Lung Adenocarcinoma	#N/A	#N/A
Lung 946	CST	Lung Adenocarcinoma	#N/A	#N/A
Stomach	BioChain	Normal Stomach	#N/A	#N/A
Stomach	BioChain	Normal Stomach	#N/A	#N/A

Vendor catalogue number, vendor, tissue sample type, and patient age and sex are listed for each of the tissues stained. "#N/A" indicates that this information was not readily available for the particular sample.

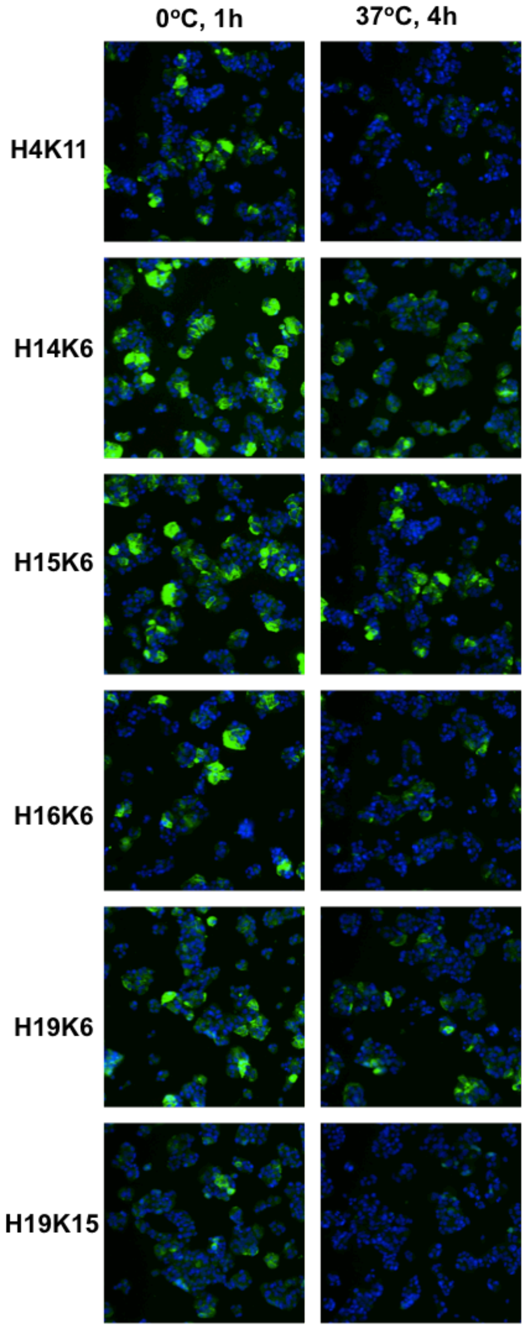

Supplementary Fig. 5. Immunofluorescence staining of MUC1 internalization following binding of human anti-MUC1 antibodies. Punctate staining is seen with H15K6 and not with the other antibodies. ZR-75-1 cells were incubated with various anti-MUC1 human antibodies for the indicated times and temperatures. Cells were then washed and fixed with

paraformaldehyde followed by detection with secondary antibodies. MUC1: anti-human IgG-Alexa 488 complexes are shown in green. Nuclei were stained with DAPI (blue).

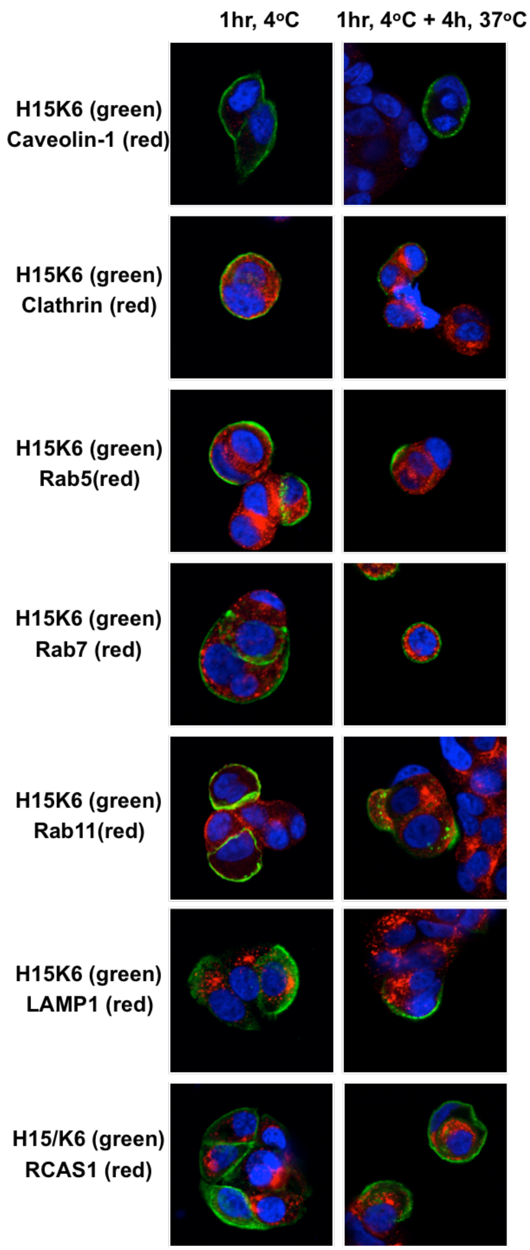

Supplementary Fig. 6. Confocal images of MUC1 internalization after binding of H15K6 and colocalization with indicated intracellular compartment markers. ZR-75-1 cells were incubated with anti-MUC1 human antibody H15K6 for the indicated times and temperatures. Cells were then washed and fixed with paraformaldehyde, then incubated with rabbit antibodies specific for molecules in the endocytic pathway (Caveolin1, Clathrin, Rab5, Rab7, Rab11), lysosome (LAMP1) or golgi (RCAS1), followed by detection with secondary antibodies (MUC1:

anti-human IgG-Alexa 488 complex is shown in green; organelle markers: anti-rabbit IgG-Alexa 555 complex is shown in red). Nuclei were stained with DAPI (blue). Signal was visualized by confocal immunofluorescence microscopy and captured images were overlaid to assess co-localization (yellow) of anti-MUC1 antibody with organelle marker antibody.

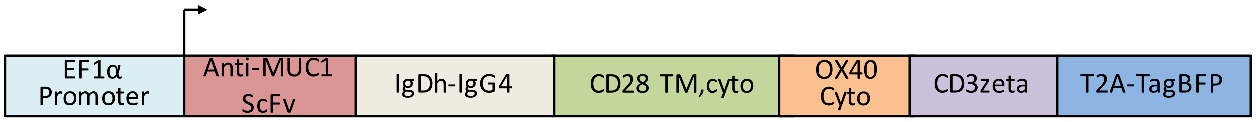

Supplementary Fig. 7. Design of CAR Lentiviral Expression Cassettes. CARs are expressed from the EF1α promoter, and consist of the scFv's of the H4K10, H4K11, H14K6, H16K6 or H19K15 antibodies fused to the IgDhinge and IgG4(CH_2,CH_3) spacer domains, the CD28 transmembrane and cytoplasmic domains, the OX40 cytoplasmic domain, CD3zeta and the T2A cleavage tag, and the TagBFP reporter gene.

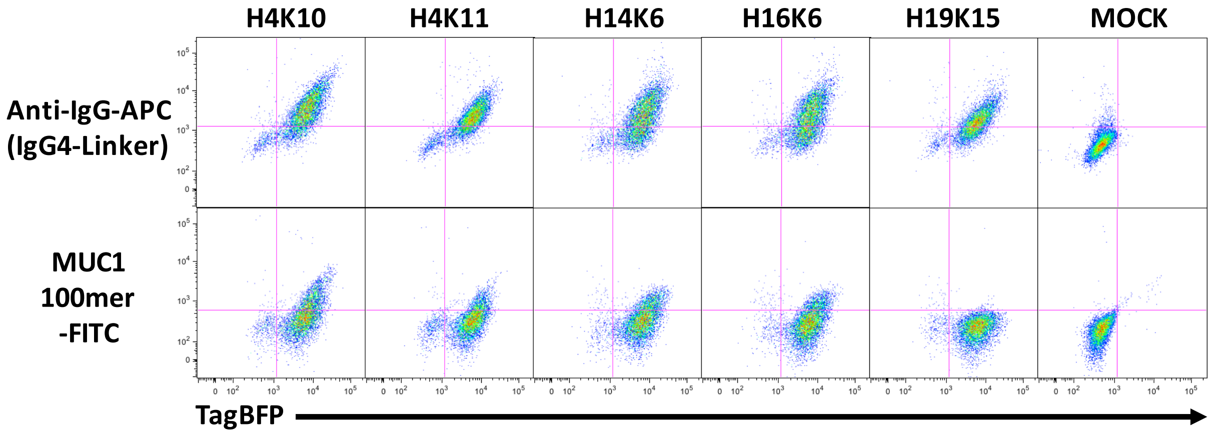

Supplementary Fig. 8. Confirmation of CAR cell surface expression by Fc-APC staining of extracellular linker domain and FITC labeled MUC1-100mer peptide. Flow sorted CAR+ cells (TagBFP+) or MOCK transduced negative control cells were stained with an anti-human IgG APC-conjugated $F(ab')_2$ to identify IgG4-linker expressing cells or FITC-labeled MUC1-100mer peptide indicating scFv binding to MUC1 peptide and evaluated by flow cytometry.

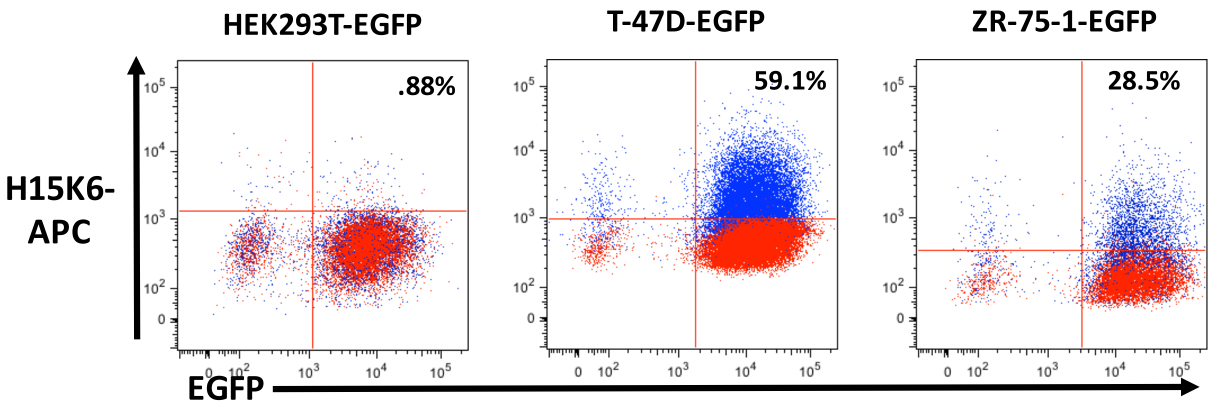

Supplementary Fig. 9. Hypoglycosylated MUC1+ populations of HEK293T-EGFP, T-47D-EGFP, and ZR-75-1-EGFP target cells. Cell lines were stained with H15K6 antibody followed by anti-human IgG APC-conjugated F(ab')$_2$ to identify the hypoglycosylated MUC1 population of each EGFP+ cell line and underwent flow cytometry. Percentage of double positive populations for APC (H15K6) and EGFP are listed in this gate for each line.

Supplementary Table 3. DNA sequences of anti-MUC1 CARs and expression vector.

3rd Generation CAR sequence for anti-MUC1 CARs
ATGGAGACAGACACACTCCTGCTATGGGTGCTGCTGCTCTGGGTTCCAGGTTCCACA
GG-scFv here-
AAAGACGGACAGCTACTACATGACAAGCAGCCAGCTCTCCACCCCCCTCCAGCAG
TGGCGCCAAGGCGAGTACAAATGCGTGGTCCAGCACACCGCCAGCAAGAGTAAGAA
GGAGATCTTCCGCTGGCCAGAGTCTCCAAAGGCACAGGCCTCCTCAGTGCCCACTGC
ACAACCCCAAGCAGAGGGCAGCCTCGCCAAGGCAACCACAGCCCCAGCCACCACCC
GTAACACAGGAAGAGGAGGAGAAGAGAAGAAGAAGGAGAAGGAGAAAGAGGAAC
AAGAAGAGAGAGAGACAAAGACACCAGAGGAGAGCAAGTACGGCCCTCCCTGCCC
CCCTTGCCCTGCCCCCGAGTTCCTGGGCGGACCCAGCGTGTTCCTGTTCCCCCCCAA
GCCCAAGGACACCCTGATGATCAGCCGGACCCCCGAGGTGACCTGCGTGGTGGTGG
ACGTGAGCCAGGAAGATCCCGAGGTCCAGTTCAATTGGTACGTGGACGGCGTGGAA
GTGCACAACGCCAAGACCAAGCCCAGAGAGGAACAGTTCAACAGCACCTACCGGGT
GGTGTCTGTGCTGACCGTGCTGCACCAGGACTGGCTGAACGGCAAAGAATACAAGT
GCAAGGTGTCCAACAAGGGCCTGCCCAGCAGCATCGAAAAGACCATCAGCAAGGCC
AAGGGCCAGCCTCGCGAGCCCCAGGTGTACACCCTGCCTCCCTCCCAGGAAGAGAT
GACCAAGAACCAGGTGTCCCTGACCTGCCTGGTGAAGGGCTTCTACCCCAGCGACA
TCGCCGTGGAGTGGGAGAGCAACGGCCAGCCTGAGAACAACTACAAGACCACCCCT
CCCGTGCTGGACAGCGACGGCAGCTTCTTCCTGTACAGCCGGCTGACCGTGGACAA
GAGCCGGTGGCAGGAAGGCAACGTCTTTAGCTGCAGCGTGATGCACGAGGCCCTGC
ACAACCACTACACCCAGAAGAGCCTGAGCCTGTCCCTGGGCAAGATGTTCTGGGTG
CTGGTGGTGGTGGGCGGGGTGCTGGCCTGCTACAGCCTGCTGGTGACAGTGGCCTTC
ATCATCTTTTGGGTGCGGAGCAAGCGGAGCAGAGGCGGCCACAGCGACTACATGAA
CATGACCCCCAGACGGCCTGGCCCCACCCGGAAGCACTACCAGCCCTACGCCCCAC
CCAGGGACTTTGCCGCCTACCGGTCCGGCGGAGGGCAGAGGCTGCCCCCCGATGCC
CACAAGCCCCCTGGGGGAGGCAGTTTCCGGACCCCCATCCAAGAGGAGCAGGCCGA
CGCCCACTCCACCCTGGCCAAGATCCGGGTGAAGTTCAGCAGAAGCGCCGACGCCC
CTGCCTACCAGCAGGGCCAGAATCAGCTGTACAACGAGCTGAACCTGGGCAGAAGG
GAAGAGTACGACGTCCTGGATAAGCGGAGAGGCCGGGACCCTGAGATGGGCGGCA
AGCCTCGGCGGAAGAACCCCCAGGAAGGCCTGTATAACGAACTGCAGAAAGACAA
GATGGCCGAGGCCTACAGCGAGATCGGCATGAAGGGCGAGCGGAGGCGGGGCAAG
GGCCACGACGGCCTGTATCAGGGCCTGTCCACCGCCACCAAGGATACCTACGACGC
CCTGCACATGCAGGCCCTGCCCCCAAGGCTCGAGGGCGGCGGAGAGGGCAGAGGA
AGTCTTCTAACATGCGGTGACGTGGAGGAGAATCCCGGCCCTCGCATGAGCGAGCT
GATTAAGGAGAACATGCACATGAAGCTGTACATGGAGGGCACCGTGGACAACCATC
ACTTCAAGTGCACATCCGAGGGCGAAGGCAAGCCCTACGAGGGCACCCAGACCATG
AGAATCAAGGTGGTCGAGGGCGGCCCTCTCCCCTTCGCCTTCGACATCCTGGCTACT
AGCTTCCTCTACGGCAGCAAGACCTTCATCAACCACACCCAGGGCATCCCCGACTTC
TTCAAGCAGTCCTTCCCTGAGGGCTTCACATGGGAGAGAGTCACCACATACGAAGA
CGGGGGCGTGCTGACCGCTACCCAGGACACCAGCCTCCAGGACGGCTGCCTCATCT
ACAACGTCAAGATCAGAGGGGTGAACTTCACATCCAACGGCCCTGTGATGCAGAAG
AAAACACTCGGCTGGGAGGCCTTCACCGAGACGCTGTACCCCGCTGACGGCGGCCT
GGAAGGCAGAAACGACATGGCCCTGAAGCTCGTGGGCGGAGCCATCTGATCGCAA
ACATCAAGACCACATATAGATCCAAGAAACCCGCTAAGAACCTCAAGATGCCTGGC
GTCTACTATGTGGACTACAGACTGGAAAGAATCAAGGAGGCCAACAACGAAACATA

CGTCGAGCAGCACGAGGTGGCAGTGGCCAGATACTGCGACCTCCCTAGCAAACTGG
GGCACAAGCTTAATTAA

Color code:

Leader sequence

IgDhinge

IgG4(CH_2,CH_3)

CD28-TM,cyto

OX40-cyto

CD3zeta

T2A-TagBFP

scFv DNA sequences (V_H-GS Linker-V_L)

H4K10

CAGCTGCAGCTGCAGGAGTCGGGCCCAGGACTGGTGAAGCCTTCGGAGACCCTGTC
CCTCACCTGCACTGTCTCTGGTGGCTCCATCAGCAGTAGTCGTTACTACTGGGGCTG
GATCCGCCAGCCCCCAGGGAAGGGGCTGGAGTGGATTGGGAGTATCTATTATAGTG
GGAGCACCTACTACAACCCGTCCCTCAAGAGTCGAGTCACCATATCCGTAGACACGT
CCAACAACCAGTTCTCCCTGAAGCTGACCTCTGTGACCGCCGCAGACACGGCTGTGT
ATTACTGTGCGAGACTCCCCGAAAGTGGGGGCTACTCGGGGTGGTTCGACCCCTGGG
GCCAGGGAACCCTGGTCACCGTCTCCTCAGGCGGTGGGAGCGGTGGTGGCTCCGGG
GGCGGTTCCGGTGGGGGCGGCAGCAGCGACATCGTGATGACCCAGTCTCCAGACTC
CCTGGCTGTGTCTCTGGGCGAGAGGGCCACCATCAACTGCAAGTCCAGCCAGAGTG
TTTTATACAGCTCCAACAATAAGAACTACTTAGCTTGGTACCAGCAGAAACCAGGAC
AGCCTCCTAAGCTGCTCATTTATTGGGCATCTACCCGGGAATCCGGGGTCCCTGACC
GATTCAGTGGCAGCGGGTCTGGGACAGATTTCACTCTCACCATCAGCAGCCTGCAGG
CTGAAGATGTGGCAGTTTATTACTGTCAGCAATATTATAATACTCCATTCACTTTCGG
CCCTGGGACCAAAGTGGAAATCAAA

H4K11

CAGCTGCAGCTGCAGGAGTCGGGCCCAGGACTGGTGAAGCCTTCGGAGACCCTGTC
CCTCACCTGCACTGTCTCTGGTGGCTCCATCAGCAGTAGTCGTTACTACTGGGGCTG
GATCCGCCAGCCCCCAGGGAAGGGGCTGGAGTGGATTGGGAGTATCTATTATAGTG
GGAGCACCTACTACAACCCGTCCCTCAAGAGTCGAGTCACCATATCCGTAGACACGT
CCAACAACCAGTTCTCCCTGAAGCTGACCTCTGTGACCGCCGCAGACACGGCTGTGT
ATTACTGTGCGAGACTCCCCGAAAGTGGGGGCTACTCGGGGTGGTTCGACCCCTGGG
GCCAGGGAACCCTGGTCACCGTCTCCTCAGGCGGTGGGAGCGGTGGTGGCTCCGGG
GGCGGTTCCGGTGGGGGCGGCAGCAGCGACATCGTGATGACCCAGTCTCCAGACTC
CCTGGCTGTGTCTCTGGGCGAGAGGGCCACCATCAACTGCAAGTCCAGCCAGAGTG
TTTTATACAGCTCCAACAATAAGAACTACTTAGCTTGGTACCAGCAGAAACCAGGAC
AGCCTCCTAAGCTGCTCATTTACTGGGCATCTACCCGGGAATCCGGGGTCCCTGACC
GATTCAGTGGCAGCGGGTCTGGGACAGATTTCACTCTCACCATCAGCAGCCTGCAGG

CTGAAGATGTGGCAGTTTATTACTGTCAGCAATATTATAGTACTCCGTTCACTTTTGG<br>CCAGGGGACCAAACTGGAGATCAAT
**H14K6**
CAGGTGCAGCTGGTGGAGTCTGGGGGAGGCTTGGTCAAGCCTGGAGGGTCCCTGAG<br>ACTCTCCTGTGCAGCCTCTGGATTCACCTTCAGTGACTACTACATGAGCTGGATCCG<br>CCAGGCTCCAGGGAAGGGGCTGGAGTGGGTTTCATACATTAGTAGTAGTGGTACTA<br>CCAAATACTACGCAGACTCTGTGAAGGGCCGATTCACCATCTCCAGGGACAACGCC<br>AAGAACTCACTGTATCTGCAAATGAACAGCCTGAGAGCCGAGGACACGGCCGTGTA<br>TTACTGTGTGAGAGAGAATTTAGGATACTGTAGTGGTGGTAGCTGCTACTACTACTA<br>CTACATGGACGTCTGGGGCAAAGGGACCACGGTCACCGTCTCCTCAGGCGGTGGGA<br>GCGGTGGTGGCTCCGGGGGCGGTTCCGGTGGGGGCGGCAGCAGCGATATTGTGATG<br>ACCCAGACTCCACTCTCCTCACCTGTCACCCTTGGACAGCCGGCCTCCATCTCCTGC<br>AGGTCTAGTCAAAGCCTCGTACACAGTGATGGAAACACCTACTTGAGTTGGCTTCAG<br>CAGAGGCCAGGCCAGCCTCCAAGACTCCTAATTTATAAGATTTCTAACCGGTTCTCT<br>GGGGTCCCAGACAGATTCAGTGGCAGTGGGGCAGGGACAGATTTCACACTGAAAAT<br>CAGCAGGGTGGAAGCTGAGGATGTCGGGGTTTATTACTGCATGCAAGCTACACAAT<br>TTCCTCTCACTTTCGGCGGAGGGACCAAGGTGGAGATCAAA
**H16K6**
CAGGTGCAGCTGGTGGAGTCTGGGGGAGGCTTGGTCAAGCCTGGAGGGTCCCTGAG<br>ACTCTCCTGTGCAGCCTCTGGATTCACCTTCAGTGACTACTACATGAGCTGGATCCG<br>CCAGGCTCCAGGGAAGGGGCTGGAGTGGGTTTCATACATTAGTAGTAGTGGTACTA<br>CCAAATACTACGCAGACTCTGTGAAGGGCCGATTCACCATCTCCAGGGACAACGCC<br>AAGAACTCACTGTATCTGCAAATGAACAGCCTGAGAGCCGAGGACACGGCCGTGTA<br>TTACTGTGTGAGAGAGAATTTAGGATACTGTAGTGGTGGGAGCTGCTTCTACTACTA<br>CTACATGGACGTCTGGGGCAAAGGGACCACGGTCACCGTCTCCTCAGGCGGTGGGA<br>GCGGTGGTGGCTCCGGGGGCGGTTCCGGTGGGGGCGGCAGCAGCGATATTGTGATG<br>ACCCAGACTCCACTCTCCTCACCTGTCACCCTTGGACAGCCGGCCTCCATCTCCTGC<br>AGGTCTAGTCAAAGCCTCGTACACAGTGATGGAAACACCTACTTGAGTTGGCTTCAG<br>CAGAGGCCAGGCCAGCCTCCAAGACTCCTAATTTATAAGATTTCTAACCGGTTCTCT<br>GGGGTCCCAGACAGATTCAGTGGCAGTGGGGCAGGGACAGATTTCACACTGAAAAT<br>CAGCAGGGTGGAAGCTGAGGATGTCGGGGTTTATTACTGCACGCAAGCTACACAAT<br>TCCCCCTCACTTTCGGCGGAGGGACCAAGGTGGAGATCAAA
**H19K15**
CAGGTGCAGCTGGTGGAGTCTGGGGGAGGCTTGGTCAAGCCTGGAGGGTCCCTGAG<br>ACTCTCCTGTGCAGCCTCTGGATTCACCTTCAGTGACTACTACATGAGCTGGATCCG<br>CCAGGCTCCAGGGAAGGGGCTGGAGTGGGTTTCATACATTAGTAGTAGTGGTACTA<br>CCAAATACTACGCAGACTCTGTGAAGGGCCGATTCACCATCTCCAGGGACAACGCC<br>AAGAACTCACTGTATCTGCAAATGAACAGCCTGAGAGCCGAGGACACGGCCGTGTA<br>TTACTGTGCGAGAGAACGAATAGGATACTGTAGTGGTGGGAGCTGCTTCTACTACTA<br>CTACATGGACGTCTGGGGCAAAGGGACCACGGTCACCGTCTCCTCAGGCGGTGGGA<br>GCGGTGGTGGCTCCGGGGGCGGTTCCGGTGGGGGCGGCAGCAGCGATATTGTGATG<br>ACCCAGACTCCACTCTCCTCACCTGTCACCCTTGGACAGCCGGCCTCCATCTCCTGC<br>AGGTCTAGTCAAAGCCTCGTACACAGTGATGGAAACACCTACTTGAGTTGGCTTCAG

CAGAGGCCAGGCCAGCCTCCAAGACTCCTAATTTATAAGATTTCTAACCGGTTCTCT<br>GGGGTCCCAGACAGATTCAGTGGCAGTGGGGCAGGGACAGATTTCACACTGAAAAT<br>CAGCAGGGTGGAAGCTGAGGATGTCGGGGTTTATTACTGCACGCAAGCTACACAAT<br>TTCCCATCACTTTCGGCCCTGGGACCAAAGTGGATATCAAA
pSICO-EF1 Lentiviral-expression vector
GTCGACGGATCGGGAGATCTCCCGATCCCCTATGGTGCACTCTCAGTACAATCTGCT<br>CTGATGCCGCATAGTTAAGCCAGTATCTGCTCCCTGCTTGTGTGTTGGAGGTCGCTG<br>AGTAGTGCGCGAGCAAAATTTAAGCTACAACAAGGCAAGGCTTGACCGACAATTGC<br>ATGAAGAATCTGCTTAGGGTTAGGCGTTTTGCGCTGCTTCGCGATGTACGGGCCAGA<br>TATACGCGTTGACATTGATTATTGACTAGTTATTAATAGTAATCAATTACGGGGTCA<br>TTAGTTCATAGCCCATATATGGAGTTCCGCGTTACATAACTTACGGTAAATGGCCCG<br>CCTGGCTGACCGCCCAACGACCCCCGCCCATTGACGTCAATAATGACGTATGTTCCC<br>ATAGTAACGCCAATAGGGACTTTCCATTGACGTCAATGGGTGGAGTATTTACGGTAA<br>ACTGCCCACTTGGCAGTACATCAAGTGTATCATATGCCAAGTACGCCCCCTATTGAC<br>GTCAATGACGGTAAATGGCCCGCCTGGCATTATGCCCAGTACATGACCTTATGGGAC<br>TTTCCTACTTGGCAGTACATCTACGTATTAGTCATCGCTATTACCATGGTGATGCGGT<br>TTTGGCAGTACATCAATGGGCGTGGATAGCGGTTTGACTCACGGGGATTTCCAAGTC<br>TCCACCCCATTGACGTCAATGGGAGTTTGTTTTGGCACCAAAATCAACGGGACTTTC<br>CAAAATGTCGTAACAACTCCGCCCCATTGACGCAAATGGGCGGTAGGCGTGTACGG<br>TGGGAGGTCTATATAAGCAGCGTTTTGCCTGTACTGGGTCTCTCTGGTTAGACCA<br>GATCTGAGCCTGGGAGCTCTCTGGCTAACTAGGGAACCCACTGCTTAAGCCTCAATA<br>AAGCTTGCCTTGAGTGCTTCAAGTAGTGTGTGCCCGTCTGTTGTGTGACTCTGGTAAC<br>TAGAGATCCCTCAGACCCTTTTAGTCAGTGTGGAAAATCTCTAGCAGTGGCGCCCGA<br>ACAGGGACTTGAAAGCGAAAGGGAAACCAGAGGAGCTCTCTCGACGCAGGACTCG<br>GCTTGCTGAAGCGCGCACGGCAAGAGGCGAGGGGCGGCGACTGGTGAGTACGCCA<br>AAAATTTTGACTAGCGGAGGCTAGAAGGAGAGAGATGGGTGCGAGAGCGTCAGTAT<br>TAAGCGGGGGAGAATTAGATCGCGATGGGAAAAAATTCGGTTAAGGCCAGGGGGA<br>AAGAAAAAATATAAATTAAAACATATAGTATGGGCAAGCAGGGAGCTAGAACGATT<br>CGCAGTTAATCCTGGCCTGTTAGAAACATCAGAAGGCTGTAGACAAATACTGGGAC<br>AGCTACAACCATCCCTTCAGACAGGATCAGAAGAACTTAGATCATTATATAATACAG<br>TAGCAACCCTCTATTGTGTGCATCAAAGGATAGAGATAAAAGACACCAAGGAAGCT<br>TTAGACAAGATAGAGGAAGAGCAAAACAAAAGTAAGACCACCGCACAGCAAGCGG<br>CCGGCCGCGCTGATCTTCAGACCTGGAGGAGGAGATATGAGGGACAATTGGAGAAG<br>TGAATTATATAAATATAAAGTAGTAAAAATTGAACCATTAGGAGTAGCACCCACCA<br>AGGCAAAGAGAAGAGTGGTGCAGAGAGAAAAAAGAGCAGTGGGAATAGGAGCTTT<br>GTTCCTTGGGTTCTTGGGAGCAGCAGGAAGCACTATGGGCGCAGCGTCAATGACGCT<br>GACGGTACAGGCCAGACAATTATTGTCTGGTATAGTGCAGCAGCAGAACAATTTGCT<br>GAGGGCTATTGAGGCGCAACAGCATCTGTTGCAACTCACAGTCTGGGGCATCAAGC<br>AGCTCCAGGCAAGAATCCTGGCTGTGGAAAGATACCTAAAGGATCAACAGCTCCTG<br>GGGATTTGGGGTTGCTCTGGAAAACTCATTTGCACCACTGCTGTGCCTTGGAATGCT<br>AGTTGGAGTAATAAATCTCTGGAACAGATTTGGAATCACACGACCTGGATGGAGTG<br>GGACAGAGAAATTAACAATTACACAAGCTTAATACACTCCTTAATTGAAGAATCGC<br>AAAACCAGCAAGAAAAGAATGAACAAGAATTATTGGAATTAGATAAATGGGCAAG<br>TTTGTGGAATTGGTTTAACATAACAAATTGGCTGTGGTATATAAAATTATTCATAAT<br>GATAGTAGGAGGCTTGGTAGGTTTAAGAATAGTTTTTGCTGTACTTTCTATAGTGAA

TAGAGTTAGGCAGGGATATTCACCATTATCGTTTCAGACCCACCTCCCAACCCCGAG
GGGACCCGACAGGCCCGAAGGAATAGAAGAAGAAGGTGGAGAGAGAGACAGAGAC
AGATCCATTCGATTAGTGAACGGATCGGCACTGCGTGCGCCAATTCTGCAGACAAAT
GGCAGTATTCATCCACAATTTTAAAAGAAAAGGGGGGATTGGGGGGTACAGTGCAG
GGGAAAGAATAGTAGACATAATAGCAACAGACATACAAACTAAAGAATTACAAAA
ACAAATTACAAAAATTCAAAATTTTCGGGTTTATTACAGGGACAGCAGAGATCCAGT
TTGGTTAGTACCGGGCCCGCTCTAGCCGTGAGGCTCCGGTGCCCGTCAGTGGGCAGA
GCGCACATCGCCCACAGTCCCCGAGAAGTTGGGGGGAGGGGTCGGCAATTGAACCG
GTGCCTAGAGAAGGTGGCGCGGGGTAAACTGGGAAAGTGATGTCGTGTACTGGCTC
CGCCTTTTTCCCGAGGGTGGGGGAGAACCGTATATAAGTGCAGTAGTCGCCGTGAAC
GTTCTTTTTCGCAACGGGTTTGCCGCCAGAACACAGGTAAGTGCCGTGTGTGGTTCC
CGCGGGCCTGGCCTCTTTACGGGTTATGGCCCTTGCGTGCCTTGAATTACTTCCACCT
GGCTGCAGTACGTGATTCTTGATCCCGAGCTTCGGGTTGGAAGTGGGTGGGAGAGTT
CGAGGCCTTGCGCTTAAGGAGCCCCTTCGCCTCGTGCTTGAGTTGAGGCCTGGCCTG
GGCGCTGGGGCCGCCGCGTGCGAATCTGGTGGCACCTTCGCGCCTGTCTCGCTGCTT
TCGATAAGTCTCTAGCCATTTAAAATTTTTGATGACCTGCTGCGACGCTTTTTTTCTG
GCAAGATAGTCTTGTAAATGCGGGCCAAGATCTGCACACTGGTATTTCGGTTTTTGG
GGCCGCGGGCGGCGACGGGGCCCGTGCGTCCCAGCGCACATGTTCGGCGAGGCGGG
GCCTGCGAGCGCGGCCACCGAGAATCGGACGGGGGTAGTCTCAAGCTGGCCGGCCT
GCTCTGGTGCCTGGCCTCGCGCCGCCGTGTATCGCCCCGCCCTGGGCGGCAAGGCTG
GCCCGGTCGGCACCAGTTGCGTGAGCGGAAAGATGGCCGCTTCCCGGCCCTGCTGC
AGGGAGCTCAAAATGGAGGACGCGGCGCTCGGGAGAGCGGGCGGGTGAGTCACCC
ACACAAAGGAAAAGGGCCTTTCCGTCCTCAGCCGTCGCTTCATGTGACTCCACGGAG
TACCGGGCGCCGTCCAGGCACCTCGATTAGTTCTCGAGCTTTTGGAGTACGTCGTCT
TTAGGTTGGGGGGAGGGGTTTTATGCGATGGAGTTTCCCCACACTGAGTGGGTGGAG
ACTGAAGTTAGGCCAGCTTGGCACTTGATGTAATTCTCCTTGGAATTTGCCCTTTTTG
AGTTTGGATCTTGGTTCATTCTCAAGCCTCAGACAGTGGTTCAAAGTTTTTTTCTTCC
ATTTCAGGTGTCGTGAGCGGCCGCTGAACTGAATTCATCGACGTTAACTATTCTAGA
GTACCCGGGCTAGGATCCTGTACAAGTAGCGGCCGCATAACTTCGTATAGTATAAAT
TATACGAAGTTATAAGCCTTGTTAACGCGCGGTGACCCTCGAGGTCGACGGTATCGA
TAAGCTCGCTTCACGAGATCATGTTTAAGGGTTCCGGTTCCACTAGGTACAATTCGA
TATCAAGCTTATCGATAATCAACCTCTGGATTACAAAATTTGTGAAAGATTGACTGG
TATTCTTAACTATGTTGCTCCTTTTACGCTATGTGGATACGCTGCTTTAATGCCTTTGT
ATCATGCTATTGCTTCCCGTATGGCTTTCATTTTCTCCTCCTTGTATAAATCCTGGTTG
CTGTCTCTTTATGAGGAGTTGTGGCCCGTTGTCAGGCAACGTGGCGTGGTGTGCACT
GTGTTTGCTGACGCAACCCCCACTGGTTGGGGCATTGCCACCACCTGTCAGCTCCTT
TCCGGGACTTTCGCTTTCCCCCTCCCTATTGCCACGGCGGAACTCATCGCCGCCTGCC
TTGCCCGCTGCTGGACAGGGGCTCGGCTGTTGGGCACTGACAATTCCGTGGTGTTGT
CGGGGAAATCATCGTCCTTTCCTTGGCTGCTCGCCTGTGTTGCCACCTGGATTCTGCG
CGGGACGTCCTTCTGCTACGTCCCTTCGGCCCTCAATCCAGCGGACCTTCCTTCCCGC
GGCCTGCTGCCGGCTCTGCGGCCTCTTCCGCGTCTTCGCCTTCGCCCTCAGACGAGTC
GGATCTCCCTTTGGGCCGCCTCCCCGCATCGATACCGTCGACCTCGATCGAGACCTA
GAAAAACATGGAGCAATCACAAGTAGCAATACAGCAGCTACCAATGCTGATTGTGC
CTGGCTAGAAGCACAAGAGGAGGAGGAGGTGGGTTTTCCAGTCACACCTCAGGTAC
CTTTAAGACCAATGACTTACAAGGCAGCTGTAGATCTTAGCCACTTTTTAAAAGAAA
AGGGGGGACTGGAAGGGCTAATTCACTCCCAACGAAGACAAGATATCCTTGATCTG

TGGATCTACCACACACAAGGCTACTTCCCTGATTGGCAGAACTACACACCAGGGCC
AGGGATCAGATATCCACTGACCTTTGGATGGTGCTACAAGCTAGTACCAGTTGAGCA
AGAGAAGGTAGAAGAAGCCAATGAAGGAGAGAACACCCGCTTGTTACACCCTGTGA
GCCTGCATGGGATGGATGACCCGGAGAGAGAAGTATTAGAGTGGAGGTTTGACAGC
CGCCTAGCATTTCATCACATGGCCCGAGAGCTGCATCCGGACTGTACTGGGTCTCTC
TGGTTAGACCAGATCTGAGCCTGGGAGCTCTCTGGCTAACTAGGGAACCCACTGCTT
AAGCCTCAATAAAGCTTGCCTTGAGTGCTTCAAGTAGTGTGTGCCCGTCTGTTGTGT
GACTCTGGTAACTAGAGATCCCTCAGACCCTTTTAGTCAGTGTGGAAAATCTCTAGC
AGCATGTGAGCAAAAGGCCAGCAAAAGGCCAGGAACCGTAAAAAGGCCGCGTTGC
TGGCGTTTTTCCATAGGCTCCGCCCCCCTGACGAGCATCACAAAAATCGACGCTCAA
GTCAGAGGTGGCGAAACCCGACAGGACTATAAAGATACCAGGCGTTTCCCCCTGGA
AGCTCCCTCGTGCGCTCTCCTGTTCCGACCCTGCCGCTTACCGGATACCTGTCCGCCT
TTCTCCCTTCGGGAAGCGTGGCGCTTTCTCATAGCTCACGCTGTAGGTATCTCAGTTC
GGTGTAGGTCGTTCGCTCCAAGCTGGGCTGTGTGCACGAACCCCCCGTTCAGCCCGA
CCGCTGCGCCTTATCCGGTAACTATCGTCTTGAGTCCAACCCGGTAAGACACGACTT
ATCGCCACTGGCAGCAGCCACTGGTAACAGGATTAGCAGAGCGAGGTATGTAGGCG
GTGCTACAGAGTTCTTGAAGTGGTGGCCTAACTACGGCTACACTAGAAGAACAGTAT
TTGGTATCTGCGCTCTGCTGAAGCCAGTTACCTTCGGAAAAAGAGTTGGTAGCTCTT
GATCCGGCAAACAAACCACCGCTGGTAGCGGTGGTTTTTTTGTTTGCAAGCAGCAGA
TTACGCGCAGAAAAAAAGGATCTCAAGAAGATCCTTTGATCTTTTCTACGGGGTCTG
ACGCTCAGTGGAACGAAAACTCACGTTAAGGGATTTTGGTCATGAGATTATCAAAA
AGGATCTTCACCTAGATCCTTTTAAATTAAAAATGAAGTTTTAAATCAATCTAAAGT
ATATATGAGTAAACTTGGTCTGACAGTTACCAATGCTTAATCAGTGAGGCACCTATC
TCAGCGATCTGTCTATTTCGTTCATCCATAGTTGCCTGACTCCCCGTCGTGTAGATAA
CTACGATACGGGAGGGCTTACCATCTGGCCCCAGTGCTGCAATGATACCGCGAGAC
CCACGCTCACCGGCTCCAGATTTATCAGCAATAAACCAGCCAGCCGGAAGGGCCGA
GCGCAGAAGTGGTCCTGCAACTTTATCCGCCTCCATCCAGTCTATTAATTGTTGCCG
GGAAGCTAGAGTAAGTAGTTCGCCAGTTAATAGTTTGCGCAACGTTGTTGCCATTGC
TACAGGCATCGTGGTGTCACGCTCGTCGTTTGGTATGGCTTCATTCAGCTCCGGTTCC
CAACGATCAAGGCGAGTTACATGATCCCCCATGTTGTGCAAAAAAGCGGTTAGCTCC
TTCGGTCCTCCGATCGTTGTCAGAAGTAAGTTGGCCGCAGTGTTATCACTCATGGTT
ATGGCAGCACTGCATAATTCTCTTACTGTCATGCCATCCGTAAGATGCTTTTCTGTGA
CTGGTGAGTACTCAACCAAGTCATTCTGAGAATAGTGTATGCGGCGACCGAGTTGCT
CTTGCCCGGCGTCAATACGGGATAATACCGCGCCACATAGCAGAACTTTAAAAGTG
CTCATCATTGGAAAACGTTCTTCGGGGCGAAAACTCTCAAGGATCTTACCGCTGTTG
AGATCCAGTTCGATGTAACCCACTCGTGCACCCAACTGATCTTCAGCATCTTTTACTT
TCACCAGCGTTTCTGGGTGAGCAAAAACAGGAAGGCAAAATGCCGCAAAAAAGGG
AATAAGGGCGACACGGAAATGTTGAATACTCATACTCTTCCTTTTTCAATATTATTG
AAGCATTTATCAGGGTTATTGTCTCATGAGCGGATACATATTTGAATGTATTTAGAA
AAATAAACAAATAGGGGTTCCGCGCACATTTCCCCGAAAAGTGCCACCTGAC
